# Effects of vitamin D supplementation on metabolic parameters, anthropometric measures, and diabetes risk in patients with prediabetes: an umbrella review of meta-analyses of randomized controlled trials

**DOI:** 10.1186/s12986-025-00994-1

**Published:** 2025-08-14

**Authors:** Roghayeh Molani-Gol, Maryam Rafraf, Sara Safari

**Affiliations:** 1 Student Research Committee, Tabriz University of Medical Sciences, Tabriz, Iran; 2 Nutrition Research Center, Department of Community Nutrition, Faculty of Nutrition and Food Science, Tabriz University of Medical Sciences, Tabriz, Iran

**Keywords:** Vitamin D, Supplementation, Prediabetes, Diabetes risk, Meta-analysis

## Abstract

**Background:**

The prevalence of prediabetes and, consequently, type 2 diabetes is increasing around the world. Previous meta-analyses reported controversial findings regarding the association between vitamin D supplementation with glycemic control and diabetes risk. This comprehensive meta-analysis summarized existing research to provide an estimate of the impact of vitamin D supplementation on metabolic parameters and diabetes risk in individuals with prediabetes.

**Method:**

A comprehensive systematic search was conducted across the Web of Science, Scopus, PubMed, and Cochrane databases, and Google Scholar using relevant keywords until 22 July 2025. The AMSTAR2 scale was used to evaluate the methodological quality of the included articles. Moreover, the certainty of the evidence was assessed using the GRADE tool. Stata 17 software was used for data analysis.

**Results:**

Fourteen meta-analyses comprising 31 randomized controlled trials (RCTs) of 3856 prediabetic patients were included in this review. Combining the findings of RCTs revealed that vitamin D supplementation significantly reduced the levels of fasting blood sugar (WMD= -0.377 mg/dl, 95% CI (-0.589, -0.165), *p* < 0.001), insulin (WMD = -0.174 µU/mL, 95% CI (-0.274, 0.074), *p* < 0.001), hemoglobin A1c (WMD = -0.479%, 95% CI (-0.714, -0.245), *p* < 0.001), and serum triglyceride (TG) (WMD = -0.385 mg/dl, 95% CI (-0.622, -0.147), *p* = 0.002) in comparison with the control group. The effects of vitamin D on insulin resistance by homeostasis model assessment, 2-hour oral glucose tolerance test plasma glucose, homeostasis model assessment of β-cell function, body mass index, and diabetes risk of participants were not significant.

**Conclusion:**

The findings of this umbrella review suggested that vitamin D supplementation could help to improve some glycemic indices and TG levels. However, due to discrepancies among the results, more well-designed RCTs are warranted to confirm and clarify the impacts of vitamin D supplementation in prediabetic patients.

**Clinical trial number:**

Not applicable.

**Graphical Abstract:**

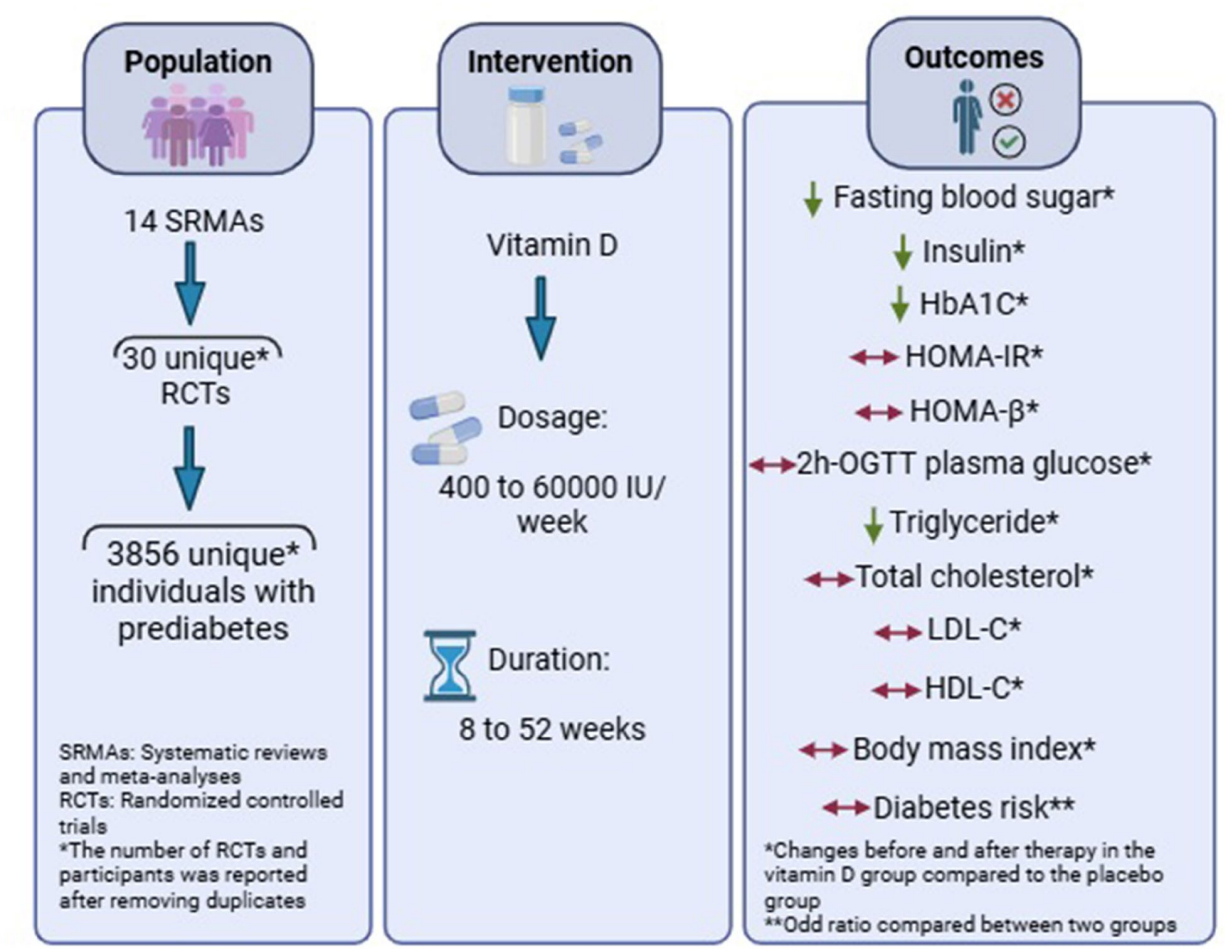

**Supplementary Information:**

The online version contains supplementary material available at 10.1186/s12986-025-00994-1.

## Introduction

Prediabetes characterized by impaired fasting glucose (IFG) or impaired glucose tolerance (IGT) [1] has become an important health concern worldwide in recent decades [2]. Prediabetic is a condition where blood glucose levels exceed normal ranges but do not meet the diabetes criteria, including fasting blood sugar (FBS) between 100 and 125 mg/dL or glycated hemoglobin (HbA1c) between 5.7% and 6.4%, or 2 h oral glucose tolerance test plasma glucose (2 h-PG) between 140 and 199 mg/dl [1]. Based on International Diabetes Federation (IDF) reports, it is estimated that 374 million adults worldwide had prediabetes in 2021 [3]. Individuals with prediabetes have a 50% higher risk of developing type 2 diabetes mellitus (T2DM) [4], and it has been shown that 70% of prediabetes cases progress to T2DM if left untreated [5]. Diabetes currently affects > 400 million individuals worldwide [6], and its prognosis has become an increasingly prevalent health problem [7]. Several vascular and non-vascular complications, including stroke, heart disease, and nerve damage, are linked to diabetes mellitus [8]. Therefore, it is essential to prevent prediabetes to diabetes progression [9]. Lifestyle changes, such as diet improvements, are the primary methods of prevention. Researchers have proposed numerous strategies to reverse prediabetes [10], with one potential approach being the use of vitamin D supplements [11, 12]. Vitamin D is a fat-soluble vitamin that plays a vital role in regulating extracellular calcium ion concentrations and maintaining the balance of calcium and phosphorus in the body [13]. Moreover, by influencing immune function, reducing inflammation and oxidation, inhibiting fibrosis, and affecting various other processes, vitamin D may lower the risk of chronic conditions such as T2DM, cardiovascular diseases, and autoimmune disorders [12, 14–17]. 

Vitamin D deficiency is a growing health problem worldwide, affecting over half of the world’s population [18]. Concurrently, research has indicated that individuals with reduced levels of serum 25-hydroxy (OH) vitamin D (25(OH)D) are more likely to exhibit elevated FBS [19] and insulin resistance [20], and a greater susceptibility to developing T2DM [21]. Numerous noncommunicable diseases, including cardiovascular disease, depression, autoimmune illnesses, osteoporosis, and diabetes, are also linked to vitamin D deficiency [22]. Nevertheless, research findings on the relationship between vitamin D and prediabetes have been inconsistent. Certain investigations have revealed that prediabetic individuals exhibit reduced serum 25(OH)D levels in comparison to those with normal glucose tolerance [23, 24]; and vitamin D deficiency faces an increased likelihood of progressing to diabetes among this population [25]. However, others demonstrated that supplementing with vitamin D failed to improve glycemic parameters in individuals with prediabetes [26]. In addition, the previous meta-analysis included a diverse number of randomized controlled trials (RCTs) and reported different results of vitamin D effects in these patients.

Therefore, there is a lack of comprehensive evidence supporting this concept, and it remains uncertain whether supplementing with vitamin D could influence the development of T2DM from prediabetes. A comprehensive systematic search and umbrella meta-analysis were performed to detect new published RCTs and summarize their results with the findings of the meta-analyses investigating the effects of vitamin D supplementation on patients with prediabetes.

## Methods

### Search strategy

This umbrella review adhered to the Preferred Reporting Items for Systematic Reviews and Meta-Analyses (PRISMA) guidelines [27] (Supplementary Table 1). The study protocol was registered on PROSPERO (registration no. CRD42024604449). A thorough literature search was conducted using the Web of Science, PubMed, Cochrane, Scopus, and ScienceDirect databases, as well as Google Scholar, without date limitations up to 22 July 2025. The search utilized MESH terms and keywords, including “vitamin D” (MESH), Calciferol, “1,25(OH)2D,” “25(OH)D,” “1,25-dihydroxy vitamin D,” and “prediabetes” (MESH). To ensure comprehensive coverage, an additional search was performed using Google, and the reference lists of included studies were examined. The complete search strategy is presented in Supplementary Table 2.

### Articles screening and selection criteria

Using EndNote 21 software, the collected studies were stored, and duplicates were eliminated. The remaining articles’ titles and abstracts were independently evaluated by two reviewers to determine which studies fell within the scope of the current review. Subsequently, the original full-text articles in English were chosen from the screened papers and thoroughly examined separately to assess their eligibility. All peer-reviewed meta-analyses that addressed the effects of vitamin D supplementation on patients with prediabetes were eligible for inclusion in this study. The PICOS (Population: prediabetes patients, Intervention: vitamin D, Comparison: placebo, Outcomes: primary outcomes including glycemic indices and secondary outcomes including lipid profiles, anthropometric parameters, and any other biochemical factors, and Study Design: meta-analyses of randomized controlled trials (RCTs)) principles were employed to establish the study’s selection criteria (Table 1). Any disagreements were addressed through consultation with an additional author. The analysis excluded various types of publications, including original articles, abstracts, conference papers, editorials, book chapters, posters, letters, and theses. Additionally, research without accessible full texts was omitted from consideration.

**Table 1:** PICOS criteria for inclusion of studies

Parameter	Inclusion criteria
Participants	Prediabetes patients or adults with impaired serum glucose levels
Intervention/correlate	Vitamin D supplementation
Comparison	Placebo or low dose of vitamin D, or control group
Outcomes	Glycemic control, anthropometric indices, lipid profile, risk of diabetes
Study design	Meta-analyses of randomized controlled trials Published in English, dated up to June 2024

### Data extraction

Two independent authors (RMG and SS) extracted the following data from the eligible studies: author name, publication year, number of participants in each meta-analysis and RCTs, study location, mean age and sex of the participants, supplement dose and duration in the RCTs, baseline 25(OH)D status of participants, number of included studies in the meta-analysis, used quality assessment tools and results, and mean (standard deviation (SD)) or odds ratio (OR) (95% confidence intervals) for the study outcomes.

### Risk of bias assessment and grading the evidence

Two independent researchers (RMG and SS) employed the AMSTAR2 (A Measurement Tool to Assess Systematic Reviews) to assess the methodological rigor of the included studies (24). This validated tool assesses each study’s pooling process and findings. This evaluation tool requires reviewers to respond to 16 questions using one of four options:

- “Yes”.
- “Partial yes”.
- “No”.
- “No meta-analysis”.

Based on these responses, the checklist categorizes the studies into four quality levels: “high,” “moderate,” “low,” and “very low.” The certainty of the evidence obtained was assessed using the GRADE tool [28]. This evaluation was based on five factors: the risk of bias, directness, consistency of results, accuracy, and publication bias. When one of these factors is not met, the quality decreases, and finally, it is classified as high, moderate, low, or very low.

### Data analysis

For this umbrella meta-analysis, due to high heterogeneity among the included studies, we employed random effects models utilizing the restricted maximum likelihood approach to determine the aggregated effect size (ES) and its corresponding 95% confidence interval. To avoid overlapping the findings of the original RCTs involved in the meta-analyses, we combined the changes in the mean and standard deviation (SD) between the intervention and placebo groups reported by the RCT studies. We followed Belbasis et al. ’s [29] recommendations for pooling the results. For assessing heterogeneity, we employed the I^2^ statistic and Cochrane’s Q test. Significant heterogeneity among studies was determined when the I^2^ value exceeded 50% or the Q test yielded a p-value less than 0.1 [30]. Subgroup analysis was based on sample size, intervention dosage, and duration, baseline serum 25(OH)D levels, year of publication, and age of the patients, which could influence the vitamin D effects in these patients. To assess the impact of removing each study on the overall effect size, we conducted a sensitivity analysis. Egger’s [31] and Begg’s [32] formal tests were used to examine the small study effect when the number of combined studies was at least 10 or less than 10, respectively. Moreover, visual inspection of the funnel plots was applied to evaluate the publication bias, and where there was an asymmetry in the funnel plot [33], to correct observed asymmetries, the “Trim-and-fill” technique was employed. A P-value < 0.05 indicates the significance of the findings. The STATA software (version 17.0; Stata Corporation, College Station, TX, USA) was utilized to conduct the statistical analyses.

## Results

### Study selection

**Fig. 1:**
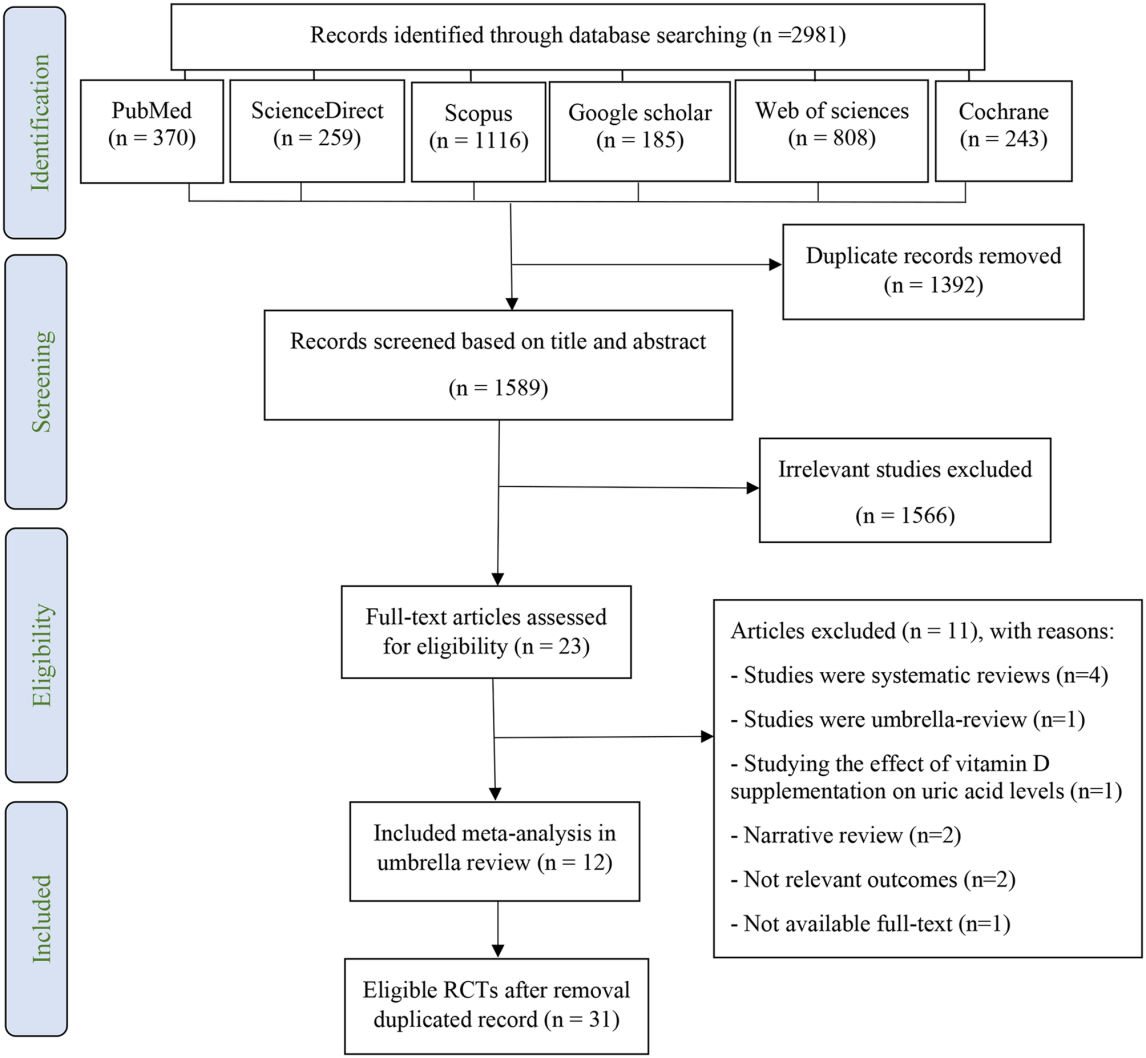
PRISMA diagram; The study selection process

Figure 1 (PRISMA diagram) illustrates the process of study search and selection for this review. A total of 2981 potential articles were identified through searches in various databases: PubMed (*n* = 370), Web of Science (*n* = 808), Scopus (*n* = 1116), Science Direct (*n* = 259), Cochrane (*n* = 243), and Google Scholar (*n* = 185). After eliminating duplicates, 1589 studies remained for further screening. Initial screening based on titles and abstracts resulted in the exclusion of 1566 irrelevant studies. The remaining 23 articles underwent critical analysis, leading to the exclusion of 11 systematic and umbrella reviews that lacked the necessary data. Supplementary Table 3 provides details on the excluded studies and the reasons for their exclusion. The final selection comprised 12 meta-analyses of RCTs, which were included in this review [9, 11, 12, 26, 34–41].

### Characteristics of the included studies

The key features of the studies included in this analysis are summarized in Table 2. The meta-analyses, published between 2012 and 2023, included five to 29 randomized controlled trials (RCTs). These studies included participants of both genders. The Cochrane Collaboration’s criteria were used to assess the quality of the RCTs, which were found to be of high standard across all meta-analyses, except for one that did not provide quality assessment information (9). After eliminating duplicate studies, the meta-analyses encompassed 31 RCTs involving 3856 prediabetic patients, with the trials conducted from 2007 to 2020. The interventions varied in dosage from 400 to 60,000 and duration between 8 and 52 weeks. Most study participants were classified as overweight or obese, with a majority exhibiting vitamin D deficiency before the intervention. A summary of the included meta-analyses’ results is presented in Supplementary Table 4.

**Table 2:** Characteristics of the included meta-analyses that examined the effects of vitamin D supplementation on prediabetic patients

First Author, year of publication/country	Population	No. of included studies	No. of included patients (intervention/placebo)	Age range (mean age)	Supplement type and dose (IU/week)	Duration (week)	Studied outcomes and Findings	Quality assessment scale and outcome
Yang et al. 2023 [40]	Prediabetics	5	601 (311/290)	20–60 years	14,000–60,000	8-260	TC, HDL, LDL↔ TG↓	Cochrane, Good
Pittas et al. 2023 [11]	Prediabetics	3	4150 (2097/2093)	61 years	20,000–28,000	NR	Risk of diabetes ↓	Cochrane, Good
Zhang et al. 2021 [38]	Prediabetics	29	3792 (2017/1780)	40–76 years	2800–60,000	8-364	BMI, 2 h-PG, HOMA-IR, and HOMA-B ↔ FBG, HbA1c, FINS (improved)	Cochrane, Good
Zou et al. 2021 [39]	Diabetics and Prediabetics	9	852	48–67 years	8400–60,000	8–48	FBS, insulin, BMI, LDL ↓ HOMA-IR, HDL↑ HbA1c, 2 h-PG, TG, TC↔	Cochrane, Good
Yu et al. 2020 [9]	Prediabetics	8	1580 (865/715)	NR	NR	NR	FBS, HOMA-IR, HbA1c ↔ 2 h-PG ↑	NR
Zhang et al. 2020 [12]	Prediabetics	8	4896	53.7 year	210-88865	24–260	The risk of T2DM ↓	Cochrane, Good
Barbarawi et al. 2020 [41]	Prediabetics	9	43,559	63.5 year	2800–88,865	1–7 years	The incidence risk of T2DM ↔	Cochrane, Good
He et al. 2018 [36]	Prediabetics	28	NR	NR	2800–60,000	NR	FPG, HOMA-IR ↔ The risk of diabetes ↓	Cochrane, Good
Mirhosseini et al. 2018 [35]	Prediabetics	28	3848	48.4 year	28,000–60,000	8 to 260	FBS, HbA1c, HOMA-IR↓	Cochrane, Good
Poolsup et al. 2016 [26]	Prediabetics	10	1671(844/827)	> 20 years	2800–88,865	8 to 364	HOMA-IR, 2 h-PG ↔ FBS, HbA1c ↓	Cochrane, Good
Seida et al. 2014 [37]	Prediabetics	5	734	52–71 years	2800–89,000	12 weeks to 7 years	HOMA-IR, HOMA-β, HbA1c, diabetes risk ↔	Cochrane, Good
George et al. 2012 [34]	Prediabetics	14	647	55.6 year	2800-120000	12 weeks to 7 years	FBS, HbA1c, insulin resistance↔	Cochrane, Good

BMI; Body mass index, FBG; Fasting blood glucose, FBS; Fasting blood sugar, 2 h-PG; 2 h oral glucose tolerance test plasma glucose, HbA1c; Hemoglobin A1c, HOMA-IR; Homeostasis model assessment of insulin resistance, HOMA-β; Homeostasis model assessment of β-cell function, FINS; Fasting insulin, NR; Not reported

### Risk of bias assessment and certainty of the evidence

The risk of bias assessment results for the included meta-analyses are presented in Supplementary Table 5. Seven meta-analyses demonstrated a low risk of bias, and five cases had a moderate risk of bias according to the AMSTAR2 criteria. Some studies failed to provide clear information regarding PICOS criteria and their protocol. The quality of the RCTs was assessed by Cochrane Collaboration’s tool, which, except for two trials, others had acceptable quality (Supplementary Table 6). Moreover, the certainty of the evidence obtained for insulin was graded as high quality, and for the others, as moderate quality, using the GRADE scale (Table 3). Additionally, regarding insulin levels, the findings of long-term trials or those with a higher intervention dose had a high certainty, whereas the results of short-term trials or those with a lower intervention dose had a moderate certainty of evidence.

**Table 3:** Summary of results and quality of evidence assessment using the GRADE approach

Outcome measures	Summary of findings		Quality of evidence assessment (GRADE)					
	**No. of patients/RCTs**	**Effect size (95% CI)**	**Risk of bias** ^**a**^	**Inconsistency** ^**b**^	**Indirectness** ^**c**^	**Imprecision** ^**d**^	**Publication bias** ^**e**^	**Quality of** **evidence** ^**f**^
Glycemic indices								
FBS	3144/25	-0.377 (-0.589, -0.165)	Not serious	Serious	Not serious	Not serious	Not serious	Moderate
Short-term trials*	1378/14	-0.189 (-0.356, -0.021)	Not serious	Serious	Not serious	Not serious	Not serious	Moderate
Long-term trials	1766/11	-0.646 (-1.044, -0.248)	Not serious	Serious	Not serious	Not serious	Not serious	Moderate
Low-dose trials**	2101/15	-0.406 (-0.665, -0.147)	Not serious	Serious	Not serious	Not serious	Not serious	Moderate
High dose trials	1043/10	-0.326 (-0.721, 0.068)	Not serious	Serious	Not serious	Not serious	Not serious	Moderate
HOMA-IR	2794/22	-0.147 (-0.496, 0.201)	Not serious	Serious	Not serious	Serious	Not serious	Moderate
Short-term trials*	1913/11	-0.181 (-0.340, -0.023)	Not serious	Not serious	Not serious	Serious	Not serious	Moderate
Long-term trials	881/11	-0.143 (-0.755, 0.469)	Not serious	Serious	Not serious	Not serious	Not serious	Moderate
Low-dose trials**	1940/13	-0.478 (-0.858, -0.098)	Not serious	Serious	Not serious	Not serious	Not serious	Moderate
High dose trials	854/9	0.384 (-0.329, 1.096)	Not serious	Serious	Not serious	Serious	Not serious	Moderate
HbA1c	3697/28	-0.479 (-0.714, -0.245)	Not serious	Serious	Not serious	Not serious	Not serious	Moderate
Short-term trials*	1828/16	-0.395 (-0.816, 0.025)	Not serious	Serious	Not serious	Serious	Not serious	Moderate
Long-term trials	1869/12	-0.569 (-0.872, -0.265)	Not serious	Serious	Not serious	Not serious	Not serious	Moderate
Low-dose trials**	2054/15	-0.373 (-0.614, -0.131)	Not serious	Serious	Not serious	Not serious	Not serious	Moderate
High dose trials	1643/13	-0.595 (-1.077, -0.113)	Not serious	Serious	Not serious	Not serious	Not serious	Moderate
2 h-PG	2310/20	-0.099 (-0.239, 0.041)	Not serious	Serious	Not serious	Serious	Not serious	Moderate
Short-term trials*	608/8	-0.066 (-0.372, 0.240)	Not serious	Serious	Not serious	Not serious	Not serious	Moderate
Long-term trials	1702/12	-0.117 (-0.277, 0.042)	Not serious	Serious	Not serious	Not serious	Not serious	Moderate
Low-dose trials**	1466/11	0.035 (-0.120, 0.190)	Not serious	Serious	Not serious	Not serious	Not serious	Moderate
High dose trials	844/9	-0.280 (-0.495, -0.064)	Not serious	Serious	Not serious	Not serious	Not serious	Moderate
HOMA-β	928/10	0.190 (-0.088, 0.468)	Not serious	Serious	Not serious	Serious	Not serious	Moderate
Short-term trials*	317/4	0.156 (-0.181, 0.492)	Not serious	Serious	Not serious	Not serious	Not serious	Moderate
Long-term trials	611/6	0.286 (-0.308, 0.879)	Not serious	Serious	Not serious	Not serious	Not serious	Moderate
Low-dose trials**	379/5	0.227 (-0.109, 0.563)	Not serious	Not serious	Not serious	Not serious	Not serious	High
High dose trials	549/5	0.183 (-0.251, 0.617)	Not serious	Serious	Not serious	Not serious	Not serious	Moderate
Insulin	2178/15	-0.174 (-0.274, -0.074)	Not serious	Not serious	Not serious	Not serious	Not serious	High
Short-term trials*	528/5	-0.125 (-0.337, 0.087)	Not serious	Not serious	Not serious	Serious	Not serious	Moderate
Long-term trials	1650/10	-0.189 (-0.307, -0.072)	Not serious	Not serious	Not serious	Not serious	Not serious	High
Low-dose trials**	1573/10	-0.124 (-0.269, 0.021)	Not serious	Not serious	Not serious	Serious	Not serious	Moderate
High dose trials	605/5	-0.271 (-0.432, -0111)	Not serious	Not serious	Not serious	Not serious	Not serious	High
Lipid profiles								
LDL-C	620/4	-0.041 (-0.239, 0.157)	Not serious	Not serious	Not serious	Serious	Not serious	Moderate
TC	620/4	-0.213 (-0.637, 0.210)	Not serious	Serious	Not serious	Serious	Not serious	Moderate
HDL-C	620/4	0.036 (-0.342, 0.413)	Not serious	Serious	Not serious	Serious	Not serious	Moderate
TG	620/4	-0.385 (-0.622, -0.147)	Not serious	Serious	Not serious	Not serious	Not serious	Moderate
Others								
BMI	2873/23	0.009 (-0.224, 0.241)	Not serious	Serious	Not serious	Serious	Not serious	Moderate
Short-term trials*	529/6	0.003 (-0.169, 0.175)	Not serious	Serious	Not serious	Not serious	Not serious	Moderate
Long-term trials	2344/17	0.021 (-0.281, 0.323)	Not serious	Serious	Not serious	Serious	Not serious	Moderate
Low-dose trials**	1561/13	0.170 (-0.197, 0.538)	Not serious	Serious	Not serious	Serious	Not serious	Moderate
High dose trials	1312/10	-0.183 (-0.461, 0.095)	Not serious	Serious	Not serious	Serious	Not serious	Moderate
Diabetes risk	1074/9	0.958 (0.889, 1.032)	Not serious	Not serious	Not serious	Serious	Not serious	Moderate
Short-term trials*	301/3	0.898 (0.527, 1.531)	Not serious	Not serious	Not serious	Serious	Not serious	Moderate
Long-term trials	773/5	0.941 (0.834, 1.062)	Not serious	Serious	Not serious	Serious	Not serious	Moderate
Low-dose trials**	669/4	0.976 (0.913, 1.043)	Not serious	Not serious	Not serious	Serious	Not serious	Moderate
High dose trials	405/4	0.720 (0.441, 1.175)	Not serious	Not serious	Not serious	Serious	Not serious	Moderate

Abbreviations: RCTs; Randomized controlled trials, TC; Total cholesterol, TG; Triglyceride, LDL-C; Low-density lipoprotein-cholesterol, HDL-C; High-density lipoprotein-cholesterol, FBS; Fasting blood sugar, BMI; Body mass index, 2 h-PG; 2 h oral glucose tolerance test plasma glucose, HbA1c; Hemoglobin A1c, HOMA-IR; Insulin resistance by homeostasis model assessment, HOMA-B; Homeostasis model assessment of β-cell function. *Shorter than 24 weeks. **Less than 30,000 IU/week

^a^ Risk of bias based on the Cochrane results

^b^ Downgraded if there was a substantial unexplained heterogeneity (I^2^ > 50%, *P* < 0.10) that was unexplained by meta-regression or subgroup analyses

^c^Downgraded if there were factors present relating to the participants, interventions, or outcomes that limited the generalizability of the results

^d^Downgraded if optimal information size was not met, or the 95%CI included the null value, lower and upper bounds of the 95%CI were < 0.95 and > 1.05, respectively

^e^Downgraded if there was evidence of publication bias using funnel plot

^f^Since all included studies were meta-analyses of randomized clinical trials, the certainty of the evidence was graded as high for all outcomes by default and then downgraded based on prespecified criteria. Certainty of the evidence was graded as high, moderate, low, very low, if 0, 1 or 2, 3 or 4, and 5 or 6 items were downgraded

### Outcomes

#### Effects of vitamin D supplementation on glycemic indices

**Fig. 2:**
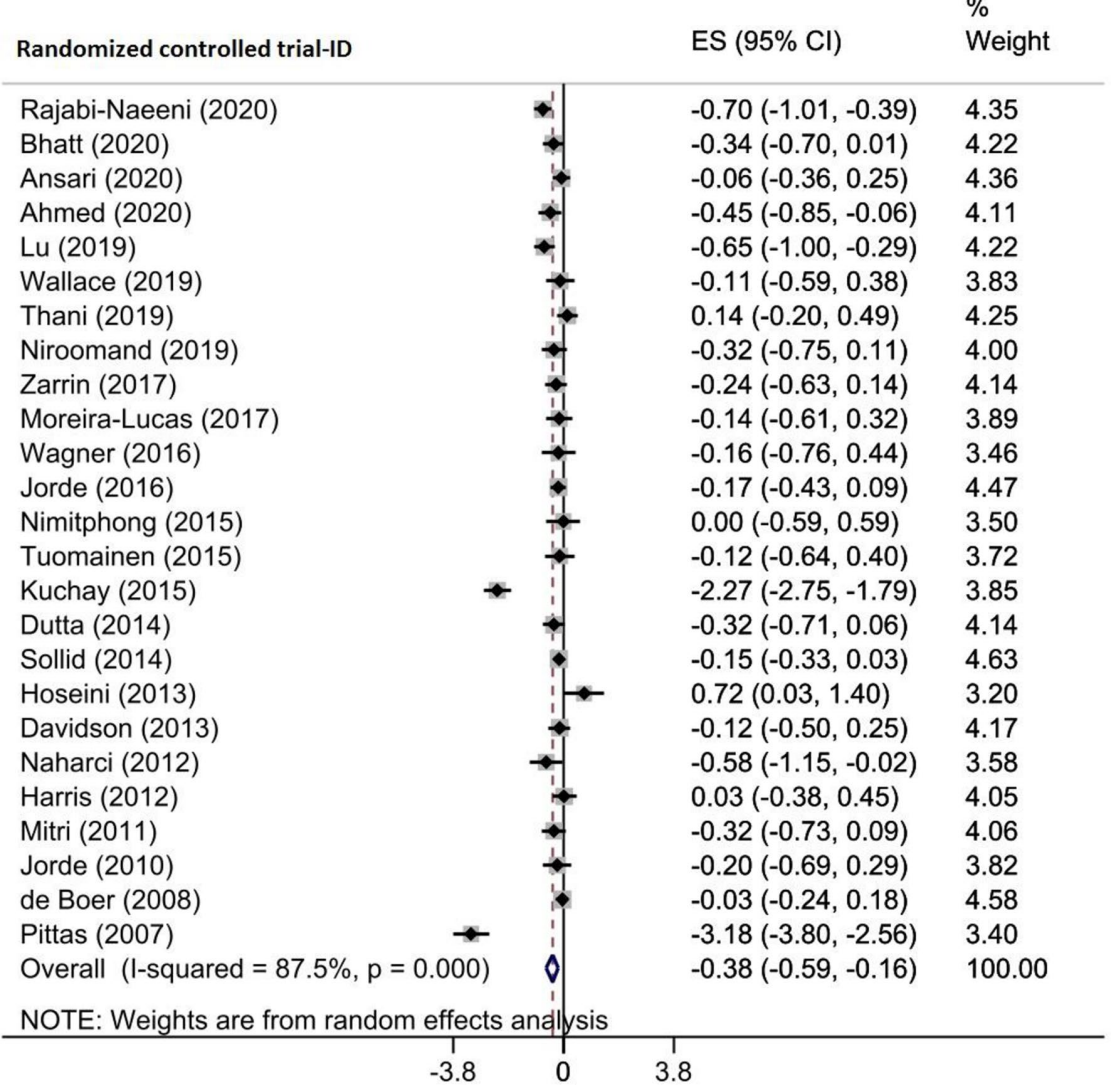
Forest plot detailing effect sizes and 95% confidence intervals for the impact of vitamin D supplementation on fasting blood sugar

Seven included meta-analyses of 25 RCTs comprising 3144 patients with prediabetes and evaluated the effects of vitamin D supplementation on serum FBS levels. Combining their findings using the random-effects model demonstrated that vitamin D intervention reduced FBS levels in participants with prediabetes (WMD= -0.377 mg/dl, 95% CI (-0.589, -0.165), *p* = 0.001) (Fig. 2). There was a high heterogeneity among the studies (I^2^ = 87.5%, P_heterogeneity_<0.001). Sub-group analysis showed that vitamin D supplementation was more effective in studies on patients aged ≥ 50 years (WMD= -0.419 mg/dl, 95% CI (-0.735, -0.103)), with sample size ≥ 100 (WMD= -0.388 mg/dl, 95% CI (-0.629, -0.147)), participants with vitamin D deficiency (WMD= -0.322 mg/dl, 95% CI (-0.460, -0.183)), and with intervention dose < 30,000 IU/week (WMD= -0.406 mg/dl, 95% CI (-0.665, -0.147)) (Supplementary Table 7). Based on the sensitivity analysis, removing each included study did not alter the results (Supplementary Fig. 1A). Egger’s results indicated no small study effect (*p* = 149); however, a partial asymmetry was observed in the funnel plot. As a result, the trim-and-fill method was used with 34 RCTs (9 imputed) in which the imputed ES increased in magnitude (WMD: -0.621 mg/dl, 95% CI (-0.872, 0.370)) (Supplementary Fig. 1B). In addition, the results of meta-regression analysis indicated that moderator variable including the sample size (*p* = 0.747), intervention dose (*p* = 0.699) and duration (*p* = 0.526), geographic region (*p* = 0.899), and baseline 25(OH)D levels (*p* = 0.733) of participants had no significant impact the vitamin D supplementation effects on FBS levels. The results of meta-regression are shown in Table 4.

**Table 4:** Meta-regression analysis results

Outcome		*N* RCTs	Coefficient	I-squared residual	*p*-value
**FBS**					
Sample size		25	0.0004	87.54%	0.747
Intervention dose		25	2.610	88.03%	0.699
Intervention duration		25	-0.001	87.93%	0.526
Geographic region		25	-0.021	87.87%	0.899
Baseline 25(OH)D status		23	0.074	88.31%	0.733
**HOMA-IR**					
Sample size		22	-0.0007	94.55%	0.791
Intervention dose		22	0.0000	94.16%	0.078
Intervention duration		22	-0.0009	94.71%	0.770
Geographic region		22	-0.443	94.78%	0.221
Baseline 25(OH)D status		21	-0.907	94.20%	0.113
**HbA1C**					
Sample size		28	0.0004	86.11%	0.742
Intervention dose		28	2.840	86.56%	0.677
Intervention duration		25	-0.001	88.01%	0.525
Geographic region		28	-0.020	86.11%	0.902
Baseline 25(OH)D status		23	0.082	88.30%	0.697
**2 h-PG**					
Sample size		20	0.0002	57.51%	0.780
Intervention dose		20	-5.620	53.82%	0.115
Intervention duration		20	0.0002	59.16%	0.847
Geographic region		20	0.117	54.11%	0.202
Baseline 25(OH)D status		20	0.227	50.75%	**0.033**
**HOMA-B**					
Sample size		10	-0.003	77.03%	0.357
Intervention dose		10	1.880	78.61%	0.789
Intervention duration		10	-0.0007	78.45%	0.892
Geographic region		10	-0.015	78.40%	0.944
Baseline 25(OH)D status		9	-0.039	71.35%	0.849
**Insulin**					
Sample size		15	-0.0001	24.37%	0.760
Intervention dose		15	-4.240	11.25%	0.177
Intervention duration		15	0.0007	0.0%	**0.043**
Geographic region		15	0.145	0.0%	**0.036**
Baseline 25(OH)D status		13	0.031	0.0%	0.687
**BMI**					
Sample size		23	-0.0000	89.20%	0.940
Intervention dose		23	-6.630	88.42%	0.249
Intervention duration		23	-0.002	87.05%	0.224
Geographic region		23	-0.052	89.03%	0.740
Baseline 25(OH)D status		20	-0.092	82.48%	0.548
**Diabetes risk**					
Sample size		8	0.0000	49.50%	0.644
Intervention dose		8	-7.240	0.0%	**0.037**
Intervention duration		8	0.001	0.0%	**0.030**
Geographic region		9	0.121	7.37%	0.153
Baseline 25(OH)D status		7	0.420	0.0%	0.079

RCT: Randomized placebo-controlled clinical trial, BMI; Body mass index, FBS; Fasting blood sugar, 2 h-PG; 2 h oral glucose tolerance test plasma glucose, HbA1c; Hemoglobin A1c, HOMA-IR; Homeostasis model assessment of insulin resistance, HOMA-B; Homeostasis model assessment of β-cell function

P-value < 0.05 is considered significant

**Fig. 3:**
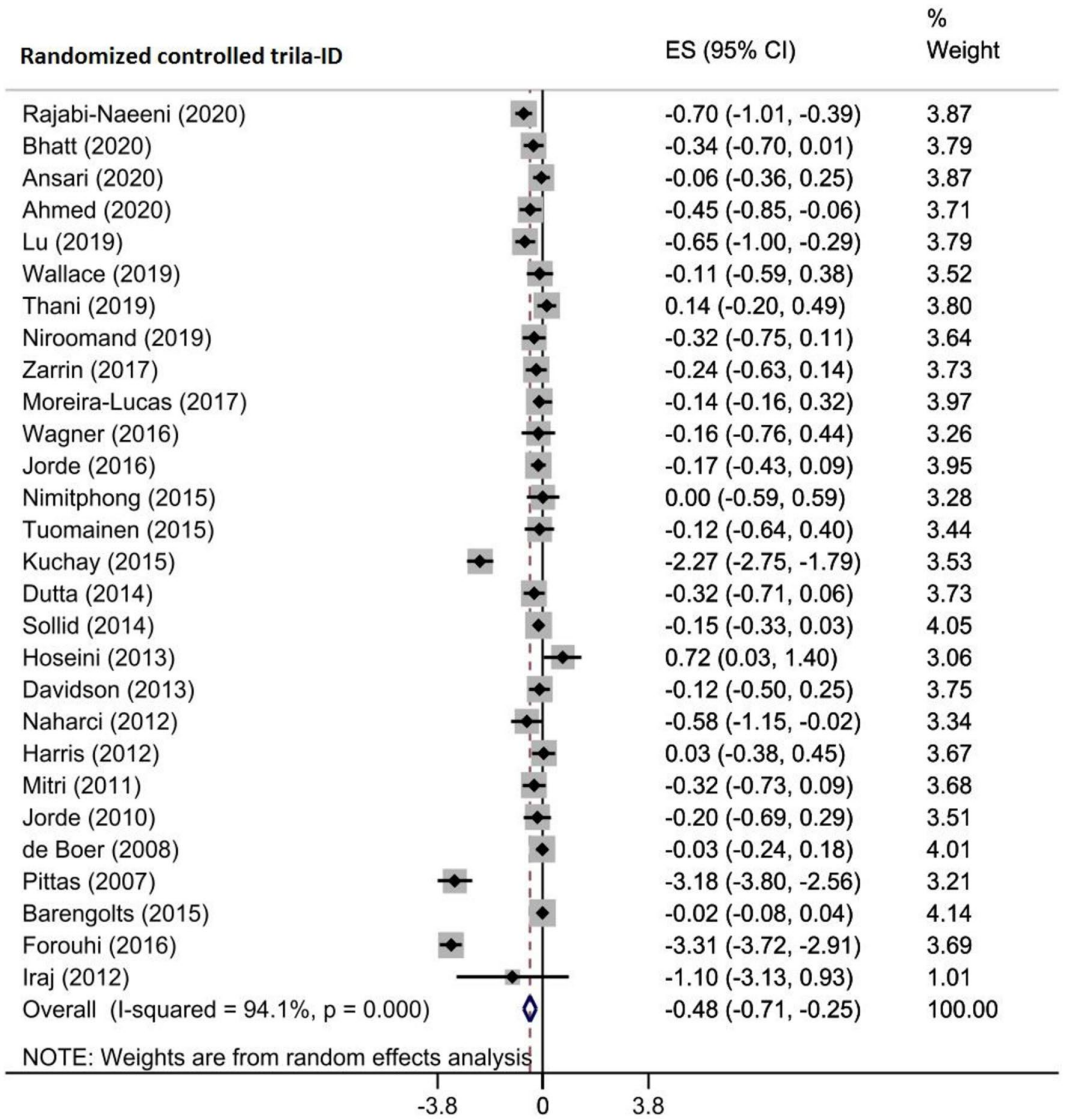
Forest plot detailing effect sizes and 95% confidence intervals for the impact of vitamin D supplementation on Hemoglobin A1c

The effects of vitamin D supplementation on HbA1c were reported by five meta-analyses that included 28 RCTs of 3697 prediabetic patients. Pooling of their findings using a random-effects model indicated a significant reduction in HbA1c after vitamin D administration (WMD = -0.479, 95% CI (-0.714, -0.245), *p* < 0.001) (Fig. 3). There was low heterogeneity among the studies (I^2^ = 94.1%, P_heterogeneity_ <0.001). In the subgroup analysis, administration of vitamin D was more effective in studies with patients aged ≥ 50 years (WMD= -0.608, 95% CI (-0.986, -0.230)), sample size ≥ 100 (WMD= -0.560, 95% CI (-0.877, -0.242)), participants with baseline vitamin D deficiency (WMD= -0.274, 95% CI (-0.453, -0.094)), and intervention duration ≥ 24 weeks (WMD= -0.569, 95% CI (-0.872, -0.265)) (Supplementary Table 7). The sensitivity analysis revealed that omitting each study alone did not change the overall effect (Supplementary Fig. 2A). According to the Egger test result, there was a significant publication bias (*p* = 0.021), and a partial publication bias was detected on the left of the funnel plot. The trim-and-fill test was applied to 37 studies (9 imputed) in which the polled ES was increased in magnitude (WMD= -0.756 (95%CI: -1.055, -0.458)) (Supplementary Fig. 2B). Meta-regression analysis also showed that the sample size (*p* = 0.742), intervention dose (*p* = 0.677) and duration (*p* = 0.525), geographic region (*p* = 0.902), and baseline 25(OH)D levels (*p* = 0.697) of patients did not influence significantly the vitamin D intervention effects on HbA1c (Table 4).

**Fig. 4:**
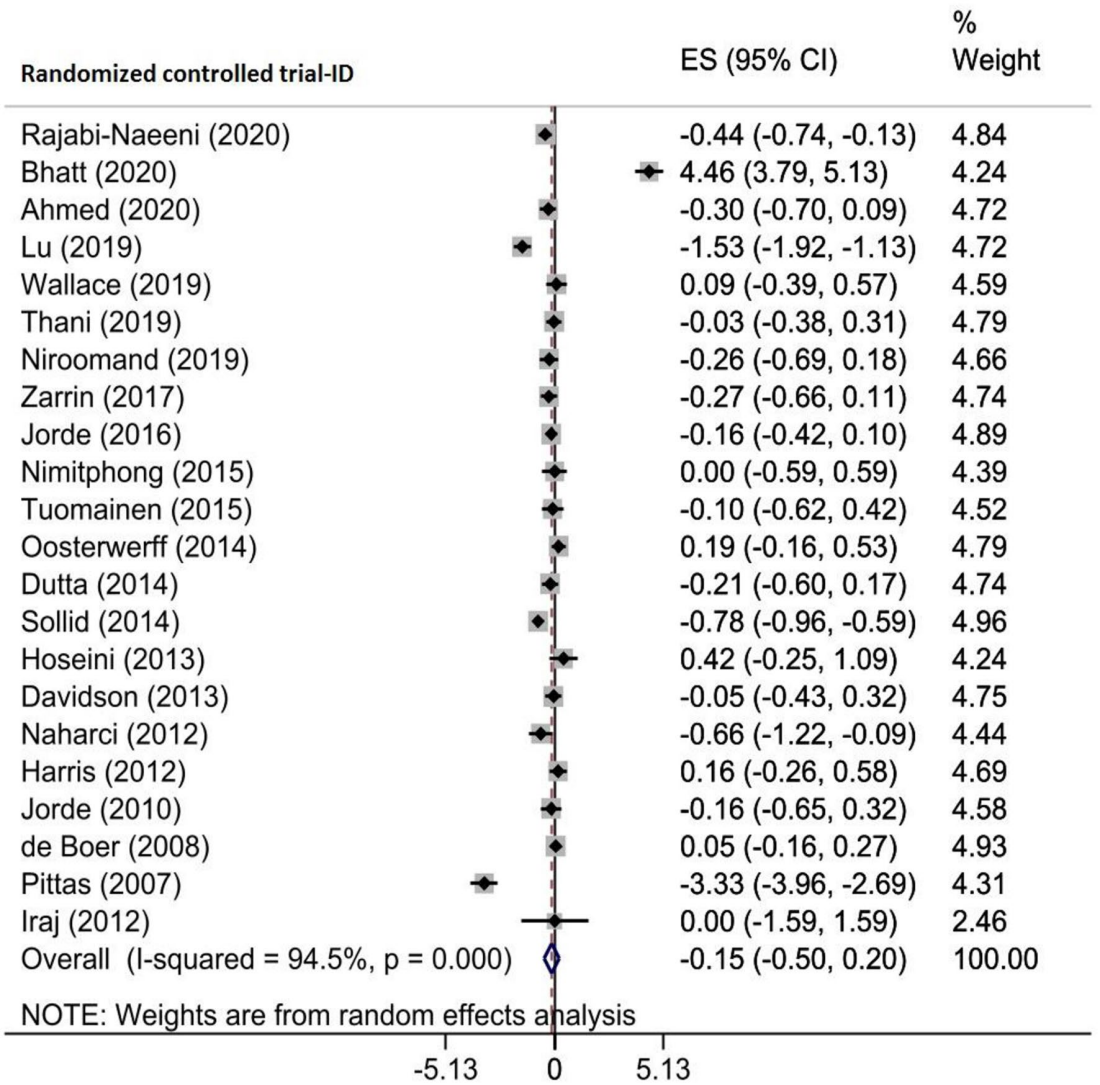
Forest plot detailing effect sizes and 95% confidence intervals for the impact of vitamin D supplementation on Homeostasis model assessment of insulin resistance (HOMA-IR)

Six meta-analyses of 22 RCTs comprising 2794 patients with prediabetes examined the effect of vitamin D supplementation on insulin resistance by homeostasis model assessment (HOMA-IR). Pooling the results from these RCTs demonstrated no significant reduction in HOMA-IR (WMD= -0.147, 95% CI (-0.496, -0.201), *p* = 0.407) with high heterogeneity among them (I^2^ = 94.5%, P_heterogeneity_<0.001) (Fig. 4). However, according to subgroup analysis, vitamin D administration significantly decreased HOMA-IR in studies of participants with sufficient and insufficient vitamin D levels (WMD = -0.739, 95% CI (-1.344, -0.135)), supplement dose < 30,000 IU/week (WMD = -0.147, 95% CI (-0.496, -0.201)), and intervention duration ≤ 24 weeks (WMD = -0.181, 95% CI (-0.340, -0.023)) (Supplementary Table 7). Based on the sensitivity analysis, after excluding the Bhatt et al. study, the pooled ES became significant (WMD = − 0.347, 95% CI: − 0.603, − 0.0921) (Supplementary Fig. 3A). The results of the Egger test showed no small study effects (*p* = 0.397). Owing to partial asymmetry in the funnel plot, the trim-and-fill test was performed without imputing any studies in which the obtained ES was not changed (Supplementary Fig. 3B). Moreover, meta-regression analysis demonstrated that moderator variables such as the sample size (*p* = 0.791), intervention dose (*p* = 0.078) and duration (*p* = 0.770), geographic region (*p* = 0.221), and baseline 25(OH)D levels (*p* = 0.113) of participants did not change significantly the vitamin D supplementation effects on HOMA-IR (Table 4).

**Fig. 5:**
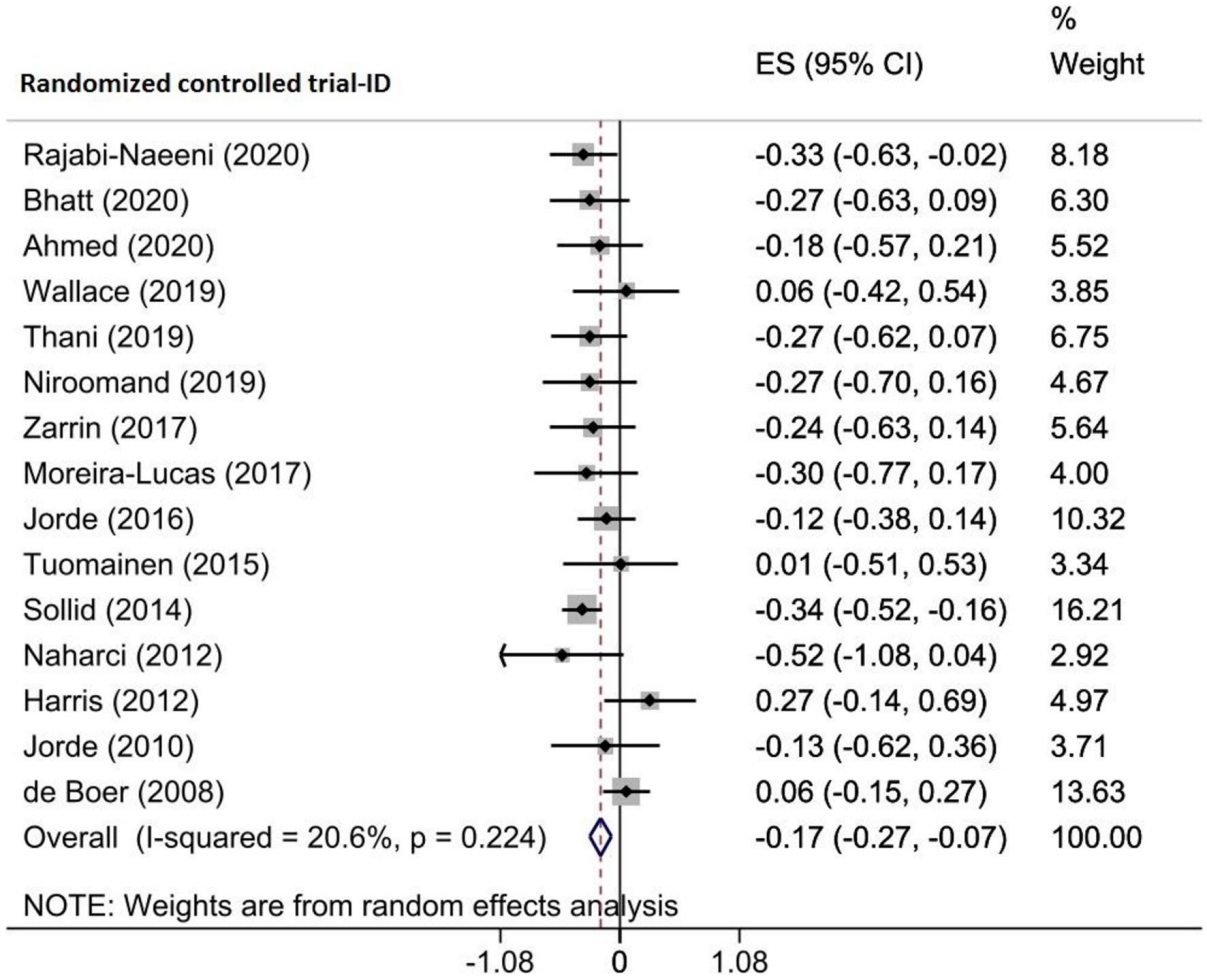
Forest plot detailing effect sizes and 95% confidence intervals for the impact of vitamin D supplementation on insulin

Four eligible meta-analyses with 15 RCTs, including 2178 prediabetic participants, examined the impact of vitamin D supplementation on insulin levels. By combining the findings of RCTs using the random-effects model, it was shown that insulin levels significantly decreased following the intervention (WMD = -0.174 µU/mL, 95% CI (-0.274, 0.074), *p* = 0.001), with a low amount of heterogeneity among RCTs (I^2^ = 20.6%, P_heterogeneity_= 0.224) (Fig. 5). Upon subgroup analysis, it was detected that vitamin D supplementation was more effective in studies with sample size ≥ 100 (WMD = -0.200 µU/mL, 95%CI (-0.317, -0.082)), published after 2015 (WMD = -0.217 µU/mL, 95% CI (-0.337, 0.097)), patients aged < 50 years (WMD = -0.270 µU/mL, 95%CI (-0.411, -0.128)), intervention dose ≥ 30,000 (WMD = -0.271 µU/mL, 95%CI (-0.432, -0.111)), and duration ≥ 24 weeks (WMD = -0.189 µU/mL, 95%CI (-0.307, -0.072)) (Supplementary Table 7). The sensitivity analysis showed that removing each study alone did not alter the obtained effect (Supplementary Fig. 4A). Egger’s test revealed no evidence of a small study effect (*p* = 0.813); moreover, visual inspection of the funnel plot did not reveal an asymmetry in the funnel plot. However, the trim-and-fill procedure was applied by imputing one trial in which the pooled ES did not change (ES: -0.162, 95% CI (-0.266, 0.057)) (Supplementary Fig. 4B). The meta-regression analysis revealed that vitamin D supplementation duration (*p* = 0.043) and geographic region of the studies (*p* = 0.036) significantly impact the vitamin D effects on insulin levels and were the possible sources of heterogeneity among the included studies. However, the sample size (*p* = 0.760), intervention dose (*p* = 0.177), and baseline 25(OH)D levels (*p* = 0.687) of participants did not relate significantly to the effect size of the study (Table 4).

**Fig. 6:**
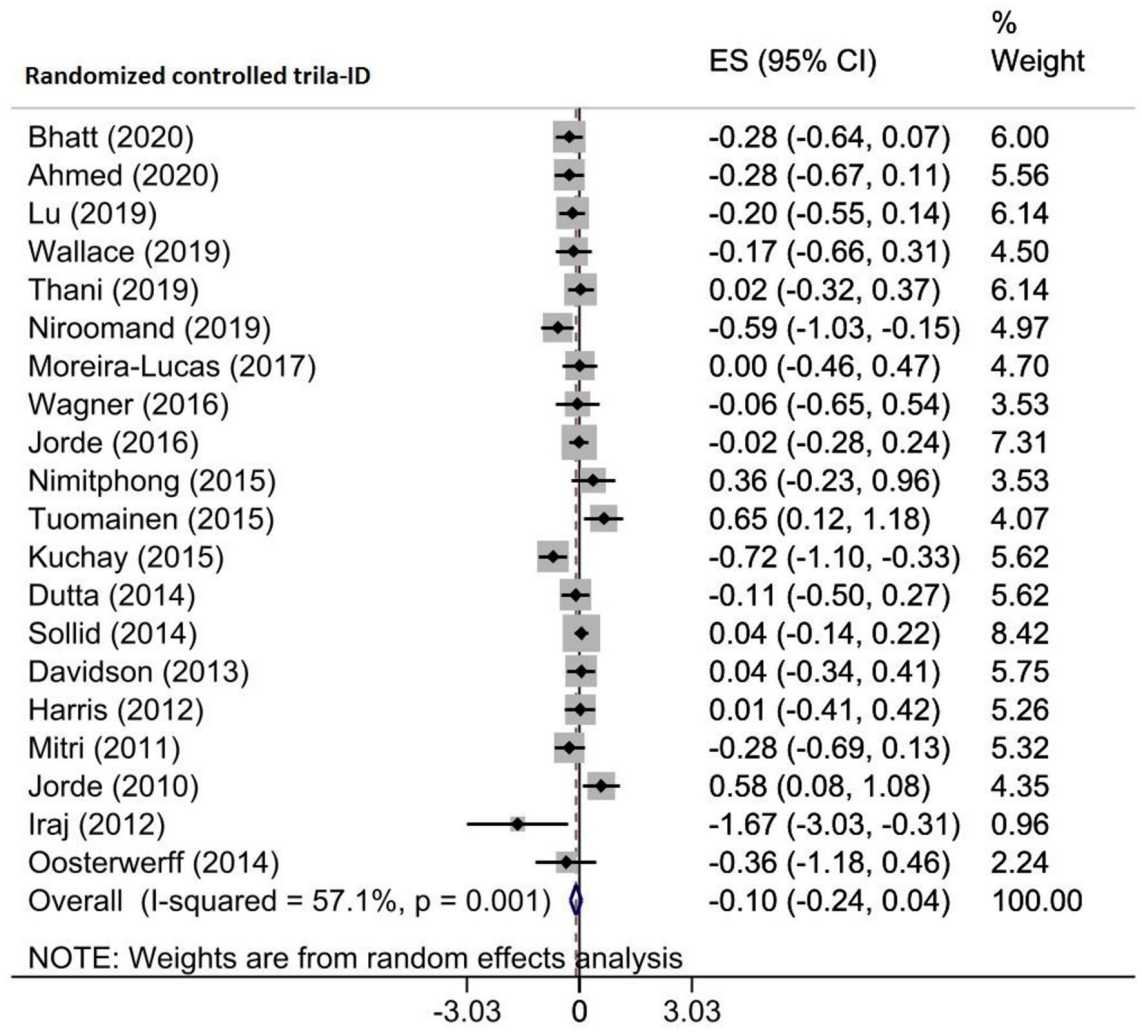
Forest plot detailing effect sizes and 95% confidence intervals for the impact of vitamin D supplementation on 2 h oral glucose tolerance test plasma glucose (2 h-PG)

The impact of vitamin D administration on 2 h-PG levels was reported by three meta-analyses, including 20 RCTs of 2310 prediabetic patients. Pooling their findings discovered that 2 h-PG levels were not considerably reduced after the intervention (WMD = -0.099 mg/dl, 95% CI (-0.239, 0.041), *p* = 0.166) (Fig. 6). There was relatively high heterogeneity among RCTs (I^2^ = 57.1%, *p* = 0.001). However, subgroup analysis revealed that the supplementation of vitamin D significantly decreased 2 h-PG in trials with sample size ≥ 100 (WMD = -0.146 mg/dl, 95%CI (-0.291, -0.002)), published after 2015 (WMD = -0.156 mg/dl, 95%CI (-0.282, -0.030)), patients aged < 50 years (WMD = -0.306 mg/dl, 95%CI (-0.574, -0.038)), participants with baseline vitamin D deficiency (WMD = -0.305 mg/dl, 95%CI (-0.519, -0.090)), and supplement dosage ≥ 30,000 IU/week (WMD = -0.305 mg/dl, 95% CI (-0.519, -0.090)) (Supplementary Table 7). According to the sensitivity analysis, by omitting each study, the results were not changed (Supplementary Fig. 5A). There was no publication bias based on Begg’s test (*p* = 0.417), but a partial asymmetry was observed in the funnel plot. As a result, the trim-and-fill method was used with 21 RCTs (one imputed) that ES was not changed significantly (ES: -0.083, 95% CI (-0.227, 0.060)) (Supplementary Fig. 5B). The results of the meta-regression analysis revealed that moderator factors such as the sample size (*p* = 0.780), intervention dose (*p* = 0.115) and duration (*p* = 0.847), geographic region (*p* = 0.202), and baseline 25(OH)D levels (*p* = 0.033) of participants did not change significantly the effects of vitamin D supplementation on 2 h-PG concentration (Table 4).

**Fig. 7:**
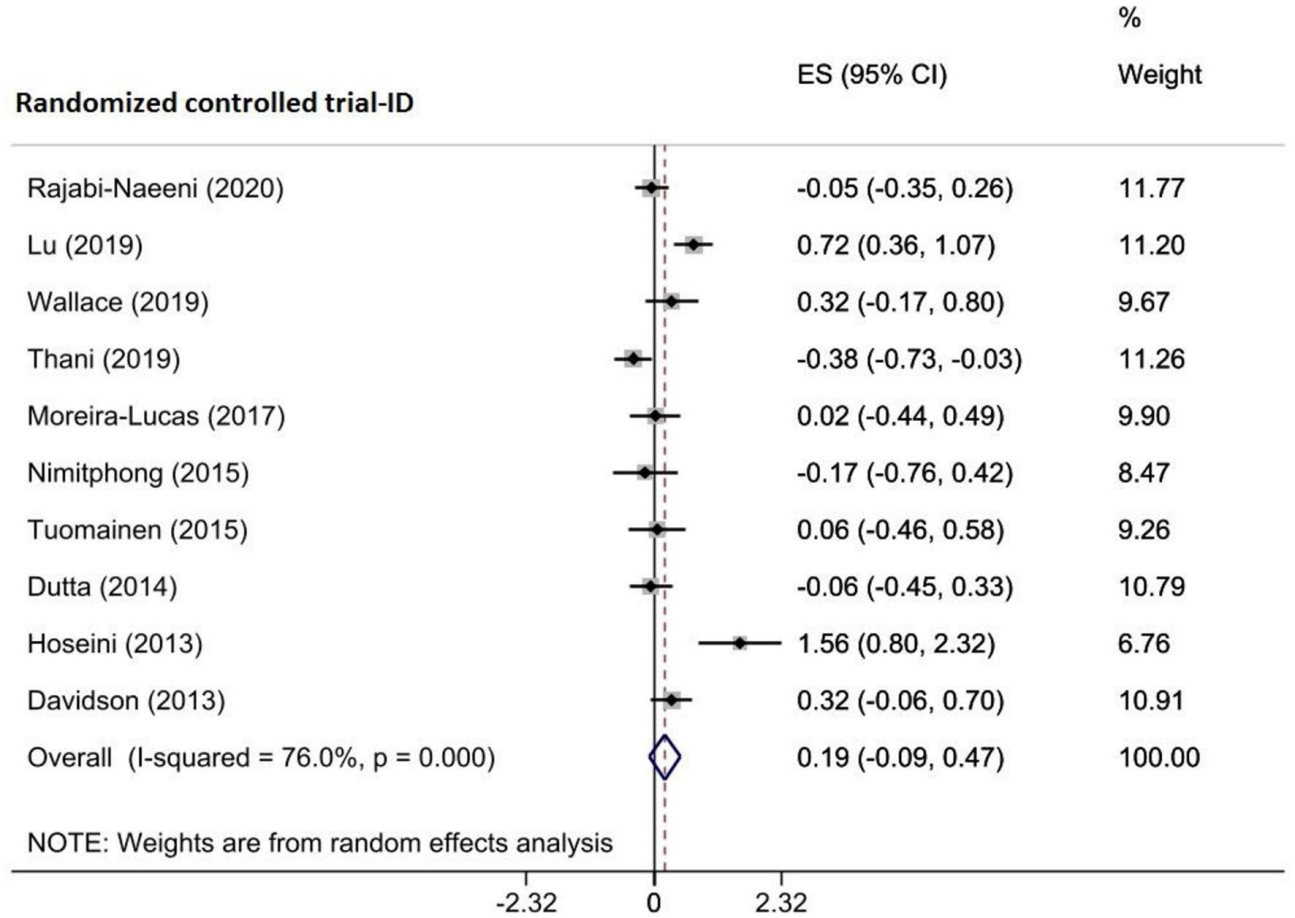
Forest plot detailing effect sizes and 95% confidence intervals for the impact of vitamin D supplementation on Homeostasis model assessment of β-cell function (HOMA-B)

Combining the findings of 10 RCTs involving 928 participants with prediabetes that were reported by three included meta-analyses indicated no significant reduction in homeostasis model assessment of β-cell function (HOMA-β) following vitamin D treatment (WMD = 0.190, 95% CI (-0.088, 0.468), *p* = 0.180) (Fig. 7). A high amount of heterogeneity was among the trials (I^2^ = 76.0%, P_heterogeneity_<0.001). Based on the subgroup analyses, sample size, intervention dose, and duration, publication year, and baseline serum vitamin D levels did not affect the pooled ES (Supplementary Table 7). By removing each included study, the obtained results did not alter in the sensitivity analysis (Supplementary Fig. 6A). Egger’s test result revealed no evidence of a minor study effect (*p* = 0.276); however, visual inspection of the funnel plot showed an asymmetry to the right of the pooled ES. Thus, the trim-and-fill test was done with 11 RCTs (one imputed), and the pooled ES was not altered (ES = 0.267, 95%CI (-0.034, 0.569)) (Supplementary Fig. 6B). Upon the meta-regression analysis, the sample size (*p* = 0.357), intervention dose (*p* = 0.789) and duration (*p* = 0.892), geographic region (*p* = 0.944), and baseline 25(OH)D levels (*p* = 0.849) of participants did not affect significantly the obtained effects (Table 4).

#### Effects of vitamin D supplementation on lipid profile

Two meta-analyses, including four trials involving 620 participants, evaluated the effects of vitamin D supplementation on serum levels of total cholesterol (TC), triglycerides (TG), low-density lipoprotein cholesterol (LDL-C), and high-density lipoprotein cholesterol (HDL-C). Combining the findings of these studies with the random-effects model indicated no significant change in serum levels of TC (WMD = -0.213 mg/dl, 95% CI (-0.637, 0.210), *p* = 0.323; I^2^ = 80.2%, P_heterogeneity_=0.002), LDL-C (WMD = -0.041 mg/dl, 95% CI (-0.239, 0.157), *p* = 0.682; I^2^ = 23.0%, P_heterogeneity_=0.270), and HDL-C (WMD = 0.036 mg/dl, 95% CI (-0.342, 0.413), *p* = 0.852; I^2^ = 78.1%, P_heterogeneity_=0.003) (Supplementary Fig. 8A, B, C) after vitamin D supplementation with a relatively high heterogeneity among the trials. While, the pooled ES of the RCTs’ findings revealed that vitamin D intervention significantly reduced serum TG levels (WMD = -0.385 mg/dl, 95% CI (-0.622, -0.147), *p* = 0.002) (Supplementary Fig. 8D). There was not considerable heterogeneity among these studies (I^2^ = 44.0%, P_heterogeneity_=0.148).

Sensitivity analyses were done, and omitting each of the RCTs did not alter the obtained results of vitamin D effects on TC, LDL-C, HDL-C, and TG levels (Supplementary Fig. 9A, B, C, D). In addition, except for LDL-C levels (*p* = 0.042), there was no publication bias for the studies of TC (*p* = 1.00), TG (*p* = 0.497), and HDL-C (*p* = 0.174) levels based on the Begg test. The trim-and-fill procedure was applied, but the pooled effect was not changed (Supplementary Fig. 10A, B, C, D).

#### Effects of vitamin D supplementation on body mass index (BMI)

**Fig. 8:**
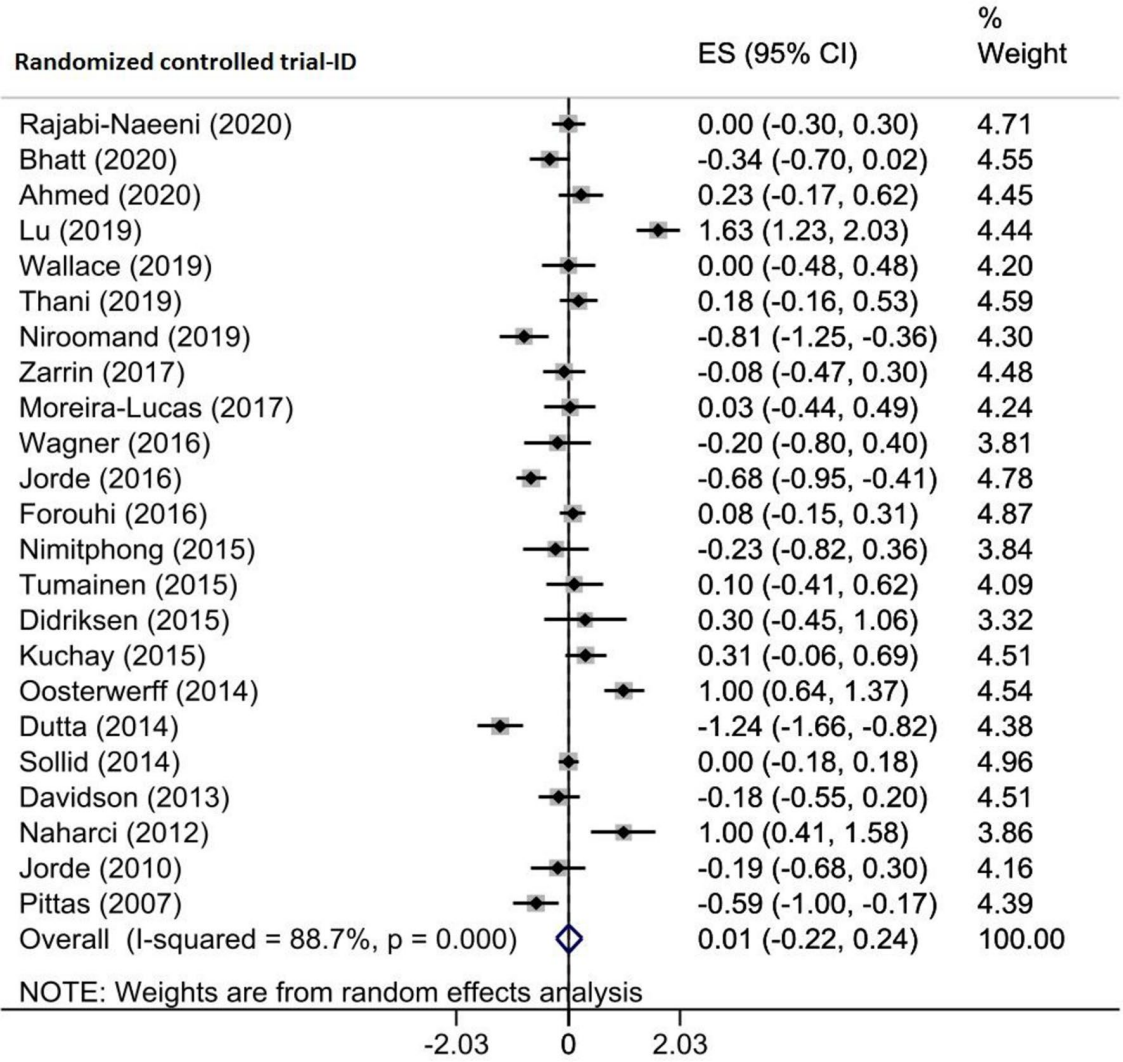
Forest plot detailing effect sizes and 95% confidence intervals for the impact of vitamin D supplementation on BMI

Three eligible meta-analyses comprising 23 RCTs of 2873 prediabetic participants investigated the effect of vitamin D supplementation on BMI. Pooling their findings using the random-effects model demonstrated that the BMI of participants did not decrease significantly after the intervention compared to the placebo group (WMD = 0.009 kg/m^2^, 95% CI (-0.224, 0.241), *p* = 0.940) (Fig. 8) with significant heterogeneity among the studies (I^2^ = 88.7%, P_heterogeneity_<0.001). Subgroup analysis revealed that the sample size, intervention dosage and duration, year of publication, baseline vitamin D levels, and age of participants among the included studies did not change the overall pooled ES (Supplementary Table 7). Based on sensitivity analysis, excluding each study did not significantly alter the results (Supplementary Fig. 7A). The Egger test indicated no significant publication bias (*p* = 0.787); however, there was an asymmetry in the funnel plot. The trim-and-fill method was conducted with 30 trials to adjust for publication bias in meta-analyses and address the asymmetry observed in funnel plots (seven imputed), and the overall effect did not alter (ES = 0.270, 95% CI (0.005, -0.535)) (Supplementary Fig. 7B). Based on the meta-regression analysis, the moderator variables such as the sample size (*p* = 0.940), intervention dose (*p* = 0.249) and duration (*p* = 0.224), geographic region (*p* = 0.740), and baseline 25(OH)D levels (*p* = 0.548) of participants had no significant influence on the vitamin D supplementation effects on BMI (Table 4).

#### Effects of vitamin D supplementation on diabetes risk

**Fig. 9:**
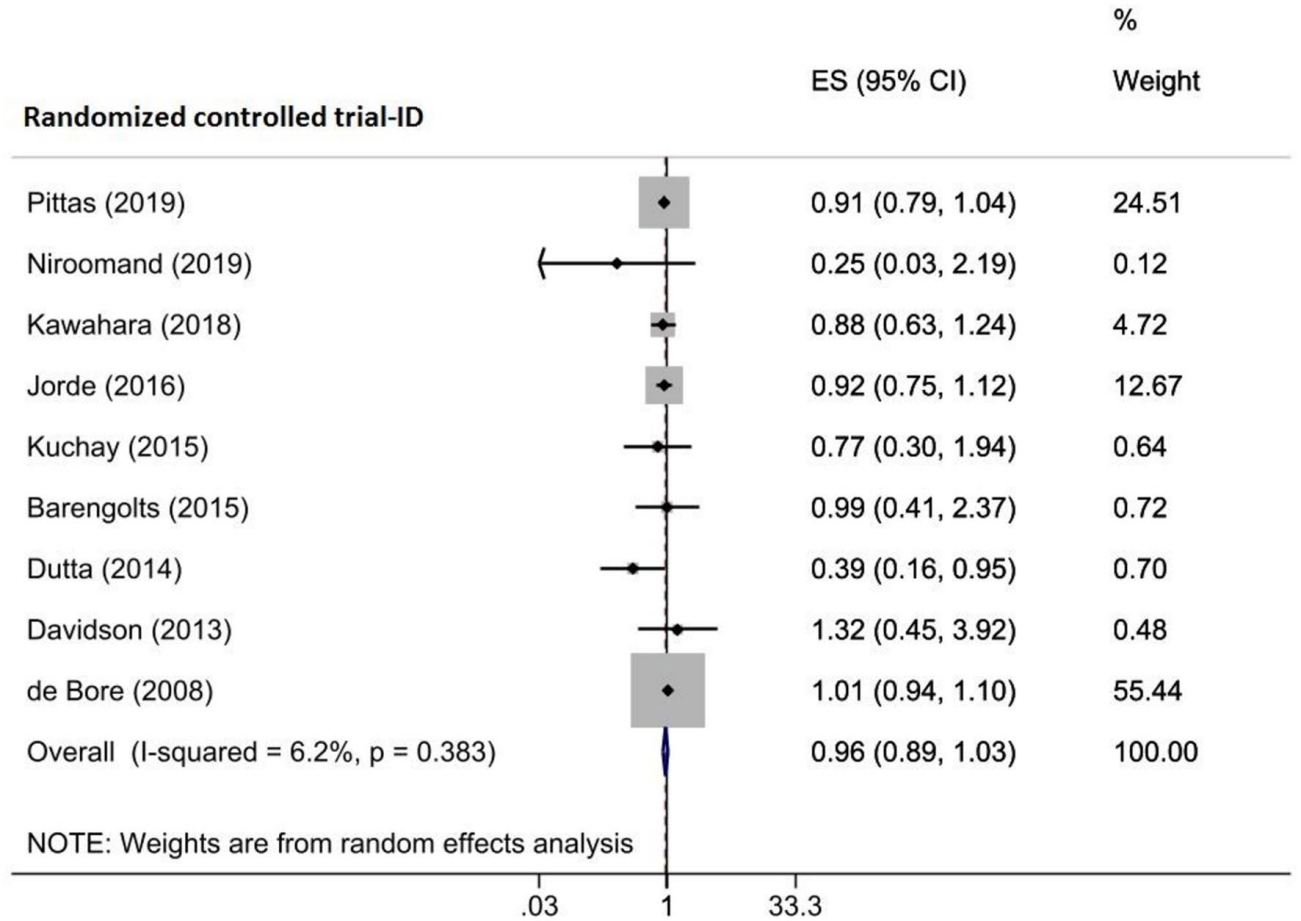
Forest plot detailing effect sizes and 95% confidence intervals for the impact of vitamin D supplementation on the risk of diabetes

Pooled ESs from nine RCTs comprising 1074 participants that were involved in five included meta-analyses indicated no significant effects of vitamin D supplementation on diabetes risk in prediabetic patients (OR = 0.958, 95%CI (0.889, 1.032)) (Fig. 9) along with a low heterogeneity between the trials (I^2^ = 6.2%, P_heterogeneity_ = 0.383). Subgroup analysis showed that the overall pooled effect was not altered with different sample sizes, baseline vitamin D levels, and intervention dosage and duration (Supplementary Table 7). Upon the sensitivity analysis, removing each trial did not change the significance of the results (Supplementary Fig. 11A). According to Begg (*p* = 0.677) and Egger (*p* = 0.071) tests’ results, there was no significant publication bias. Based on the visual inspection, there was an asymmetry in the funnel plot, so the trim-and-fill was done with three imputed studies, and the overall effect was not changed (OR = 0.966, 95% CI (0.896, 1.042)) (Supplementary Fig. 11B). Based on the meta-regression analysis, vitamin D intervention dose (*p* = 0.037) and duration (*p* = 0.030), as likely sources of heterogeneity, significantly moderated the vitamin D effects on HOMA-β. Nevertheless, the sample size (*p* = 0.644), geographic region (*p* = 0.153), and baseline 25(OH)D levels (*p* = 0.079) of participants did not significantly connect with the vitamin D supplementation effects on (Table 4).

## Discussion

The present umbrella meta-analysis comprehensively summarizes the findings of previous meta-analyses of 31 RCTs regarding the effects of vitamin D supplementation on glycemic indices, anthropometric measures, lipid profiles, and the risk of T2DM in 3856 patients with prediabetes. This meta-analysis revealed that administering vitamin D in prediabetic patients decreased levels of FBS, insulin, HbA1C, and serum TG compared to the control group. However, the effects of vitamin D intervention on HOMA-IR, 2 h-PG, HOMA-β, TC, LDL-C, HDL-C, BMI, and diabetes risk of participants were not significant. Nevertheless, vitamin D administration significantly decreased 2 h-PG when the intervention dosage was 30,000 IU/week or higher, participants’ age was under 50 years, and patients had vitamin D deficiency before the intervention. Vitamin D was more effective in trials with intervention doses of more than 30,000 IU/week, administration duration longer than 24 weeks, and participants with vitamin D deficiency and under 50 years old. The majority of the included trials were of moderate quality, and most of the evidence was rated moderate certainty, which could be reliable for clinical implications.

The findings suggest that vitamin D intervention in prediabetic patients could improve glucose metabolism, and its high dose helps relieve insulin resistance in patients with vitamin D deficiency. Several potential mechanisms of action may elucidate the possible roles of vitamin D, including its effects on immune modulation and reduction of inflammation [42, 43], as well as its ability to stimulate insulin production and release by pancreatic β-cells [44, 45]. The vitamin D receptor [46], 1-α hydroxylase [47], and vitamin D-binding protein [48] are present in pancreatic islet cells. Vitamin D enhances islet-cell secretory function by modulating the local pancreatic islet renin-angiotensin system [49]. Furthermore, vitamin D might decrease insulin resistance in peripheral insulin-target cells through the vitamin D receptor found in adipocytes, muscle [44], and hepatocytes [50] while also promoting the expression of insulin receptors and improving insulin responsiveness for glucose transport [51]. Vitamin D also indirectly affects insulin secretion by pancreatic β-cells and insulin-mediated intracellular processes through its regulation of calcium concentration [44]. It can affect gene regulation in cell proliferation, differentiation, and apoptosis within metabolic pathways [45]. Moreover, a deficiency in vitamin D can lead to a moderate increase in parathyroid hormone (PTH), which may hinder insulin release from pancreatic β-cells [52]. Additionally, vitamin D contributions were also indicated in further diseases and human life [53, 54].

The findings suggest that supplementing with vitamin D may lower triglyceride (TG) levels in individuals with prediabetes. This effect could be achieved through various mechanisms, including: elevating calcium levels, inhibiting PTH release, reducing lipolysis and inflammation, decreasing renin-angiotensin-aldosterone system (RAAS) activity, interacting with glucocorticoids and sex hormones, increasing adiponectin production, enhancing insulin sensitivity and levels, suppressing the expression of nuclear factor sterol regulatory element-binding protein 1c (SREBP1c) involved in hepatic TG production, boosting TG clearance by upregulating lipoprotein lipase (LPL), neutral sphingomyelinases, peroxisome proliferator-activated receptor gamma (PPARγ), and adipocyte-binding protein 2 (AP2), or by enhancing mitochondrial oxidation [55–61].

This study showed that vitamin D treatment did not considerably reduce the patients’ BMI. Similarly, a recent meta-analysis by Oussaada et al. demonstrated that vitamin D treatment does not significantly reduce anthropometric indices [62]. In addition, the findings suggest that vitamin D could not decrease the risk of T2DM in prediabetic patients. Some large studies also indicated that treatment with a vitamin D analog was not associated with a reduction in the incidence of T2DM [63]. Several factors may prevent prediabetes progression to diabetes, such as novel anthropometric indices [64, 65]. On the other hand, some factors may confound the influence of vitamin D supplements in these patients. The majority of participants in the RCTs were overweight or obese. There is a hypothesis that in obese individuals, 1,25-dihydroxyvitamin D (1,25(OH)D) concentration is high, which can act to limit the production of its precursor and reduce the levels of 25(OH)D [66]. In obese individuals, the fat tissue located beneath the skin exhibits reduced levels of CYP2R1, an enzyme crucial for vitamin D 25-hydroxylation. Additionally, there is a potential decrease in the expression of 1-α hydroxylase in obese subjects [66]. These findings indicate that obesity may impair both the 25-hydroxylation and 1-α hydroxylation processes of vitamin D metabolism [66]. Moreover, it is demonstrated that the percentage of body fat and total fat mass has an inverse association with 25(OH)D levels regardless of age, latitude, and longitude [52]. Vitamin D, as a fat-soluble substance, preferentially deposits in body fat, decreasing vitamin D supplement bioavailability and consequently the potential effects [67]. A meta-analysis also showed that vitamin D supplementation in T2DM patients showed more improvement in glycemic markers among non-obese individuals than obese individuals [68]. The researchers propose that obese individuals, even those with insufficient vitamin D levels, may not gain additional benefits from supplementation due to the potential storage of some supplemented doses in their adipose tissue [68]. It is proposed that obese people’s response to vitamin D supplementation is approximately 30% lower compared to non-obese individuals, and vitamin D replacement therapy should be tailored based on body size to reach the desired serum 25(OH)D levels [69]. Consequently, vitamin D supplementation might not have produced the expected effects on the examined glycemic parameters because of variations in vitamin D metabolism resulting from differences in BMI and body fat. Additionally, the diverse ethnic backgrounds of the populations across different studies could have impacted the outcomes [70]. Furthermore, vitamin D administration may play a role in preventing T2DM in high-risk populations in higher doses or intervention durations. Vitamin D treatment could be more effective in patients under 50 years, as these populations possibly have fewer metabolic disorders. Overall, multiple mechanisms provide a foundation for ongoing interest among researchers in the clinical field to explore the potential roles of vitamin D supplements in enhancing glucose metabolism and lowering the risk of T2DM.

### The strengths and limitations

The are some strengths and limitations for this study. The present umbrella meta-analysis, which had a comprehensive systematic search and was conducted by two independent authors, presented reliable evidence. Moreover, to avoid overlapping the results of original RCTs included in the meta-analyses, we combined the findings of RCTs rather than the meta-analyses. However, there was heterogeneity among the RCTs regarding doses of vitamin D, intervention duration, baseline vitamin D status of the participants, ethnic and geographical region, and weight status of the patients.

## Conclusion

The findings of the present umbrella review suggest that vitamin D supplementation could decrease levels of FBS, insulin, HbA1C, and serum TG in prediabetic patients but fail to reduce HOMA-IR, 2 h-PG, HOMA-B, BMI, and diabetes risk in these patients. Nevertheless, the clinical trials included in the study showed inconsistent outcomes. To validate these results and further investigate the impact of vitamin D supplementation on individuals with prediabetes, more extensive and well-structured randomized controlled trials are necessary. This is particularly important for overweight or obese patients who are deficient in vitamin D.

## Supplementary Information

Below is the link to the electronic supplementary material.

Supplementary Material 1

Supplementary Material 2

## Data Availability

No datasets were generated or analysed during the current study.
