# Supplementary material for: Effects of vitamin D supplementation on metabolic parameters, anthropometric measures, and diabetes risk in patients with prediabetes: an umbrella review of meta-analyses of randomized controlled trials
Source: Nutr Metab (Lond). 2025 Aug 14;22:99. doi: 10.1186/s12986-025-00994-1 (PMC12351829; doi:10.1186/s12986-025-00994-1)
Supplement: Supplementary file 1 — Supplementary Material 1 [file 12986_2025_994_MOESM1_ESM.docx]

**A**


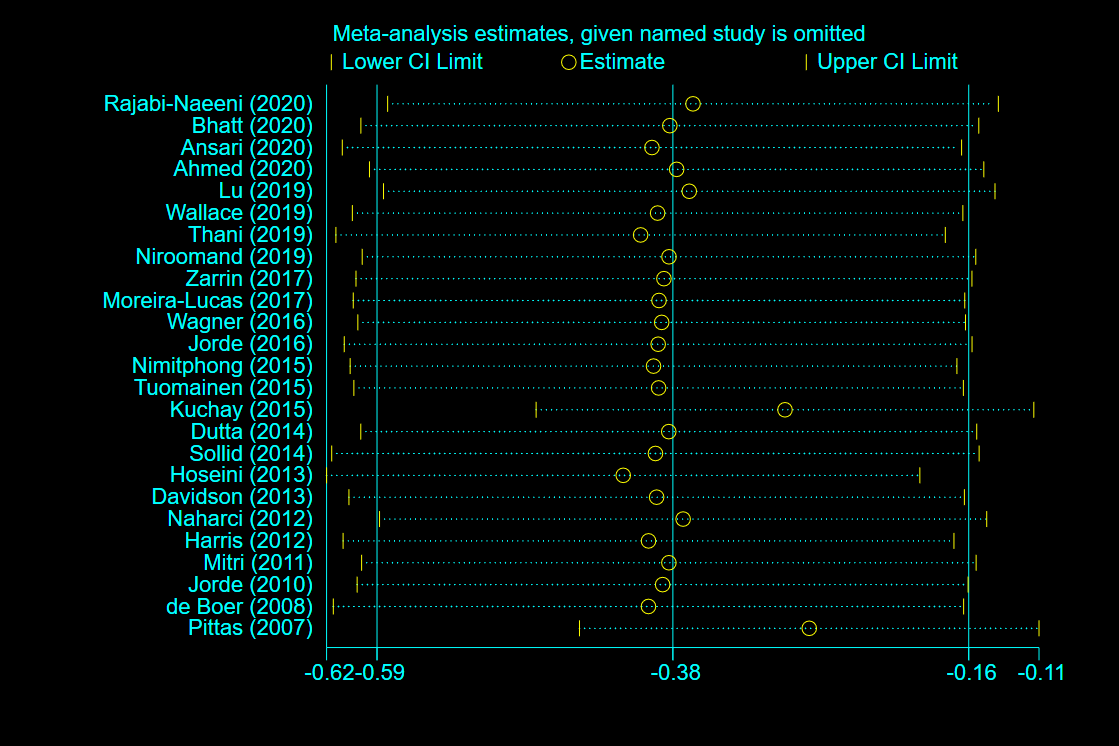


**B**

**
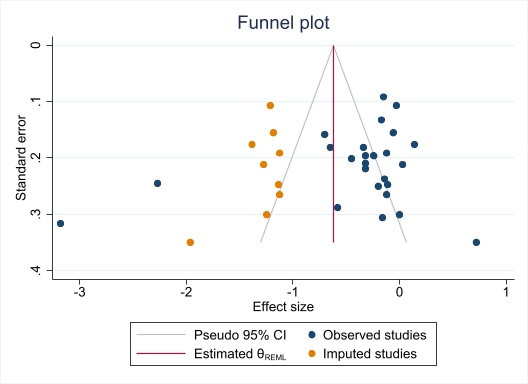
Supplementary Fig. 1**. The results of sensitivity analysis meta-analyses of vitamin D effects on fasting blood sugar (A) and the trim-and-fill method results (B)

**A**


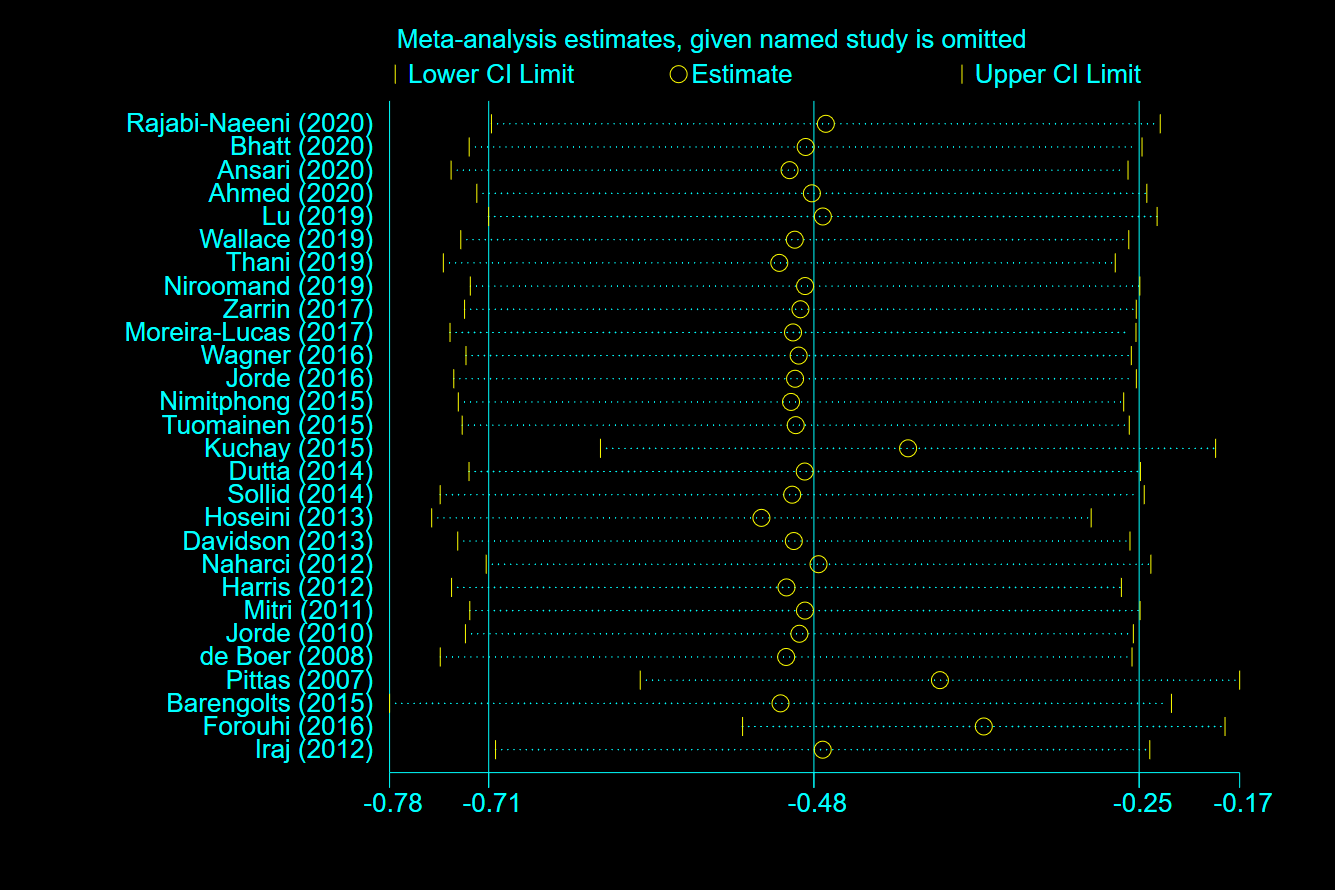


**B**

**
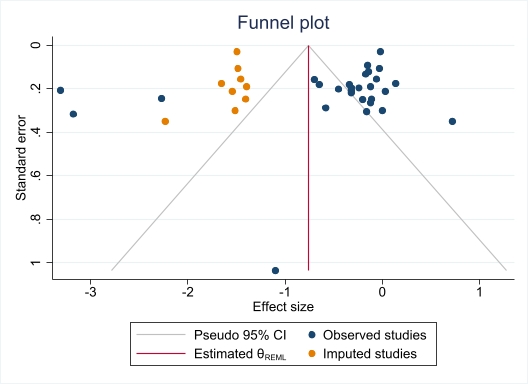
**

**Supplementary Fig. 2**. The results of sensitivity analysis meta-analyses of vitamin D effects on HbA1C (A) and the trim-and-fill method results (B)

**A**


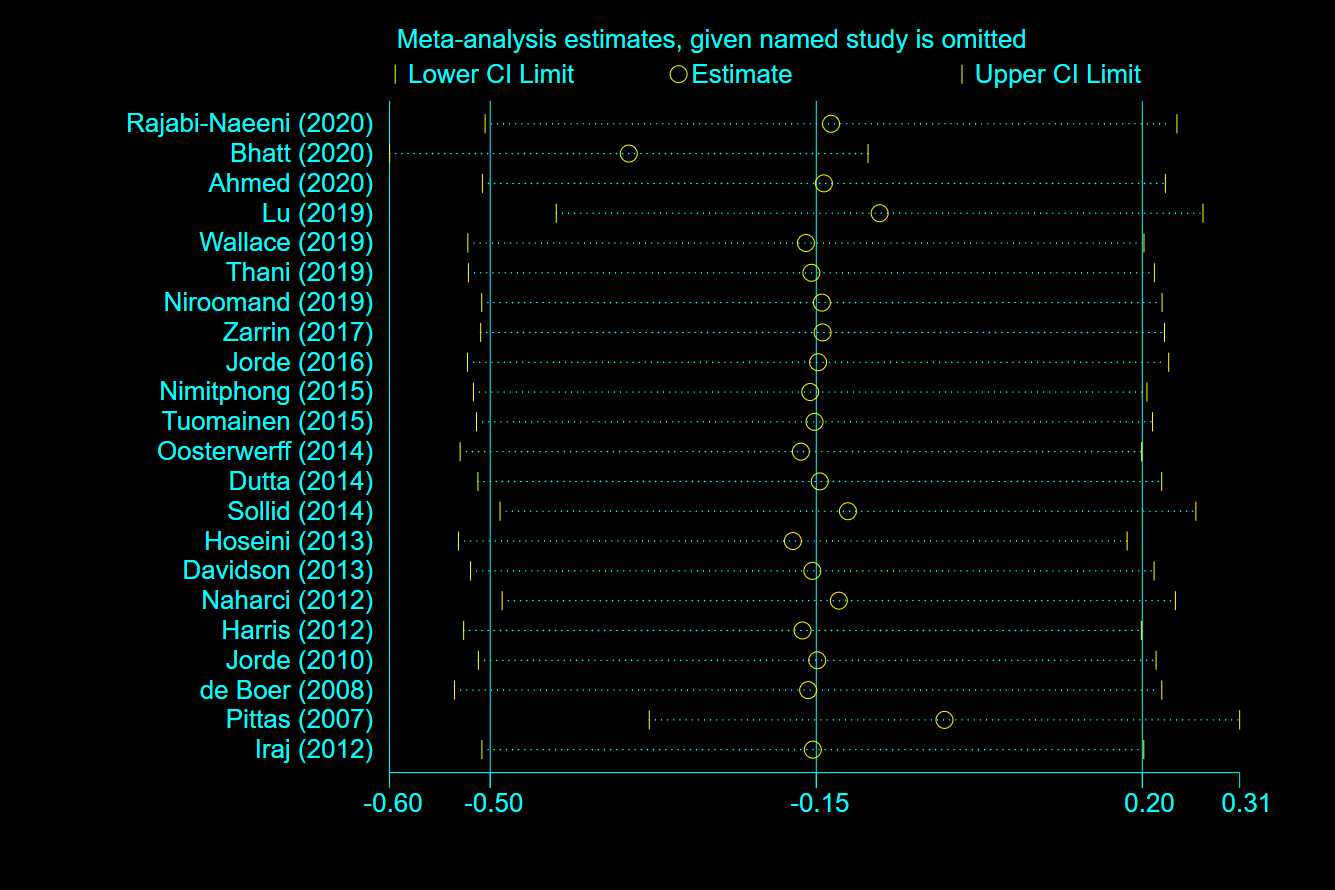


**B**

**
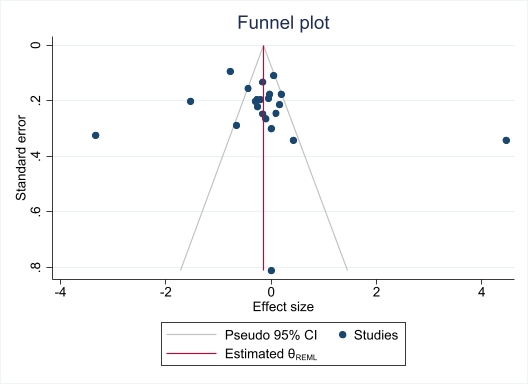
**

**Supplementary Fig. 3**. The results of sensitivity analysis meta-analyses of vitamin D effects on HOMA-IR (A) and the trim-and-fill method results (B)

**A**


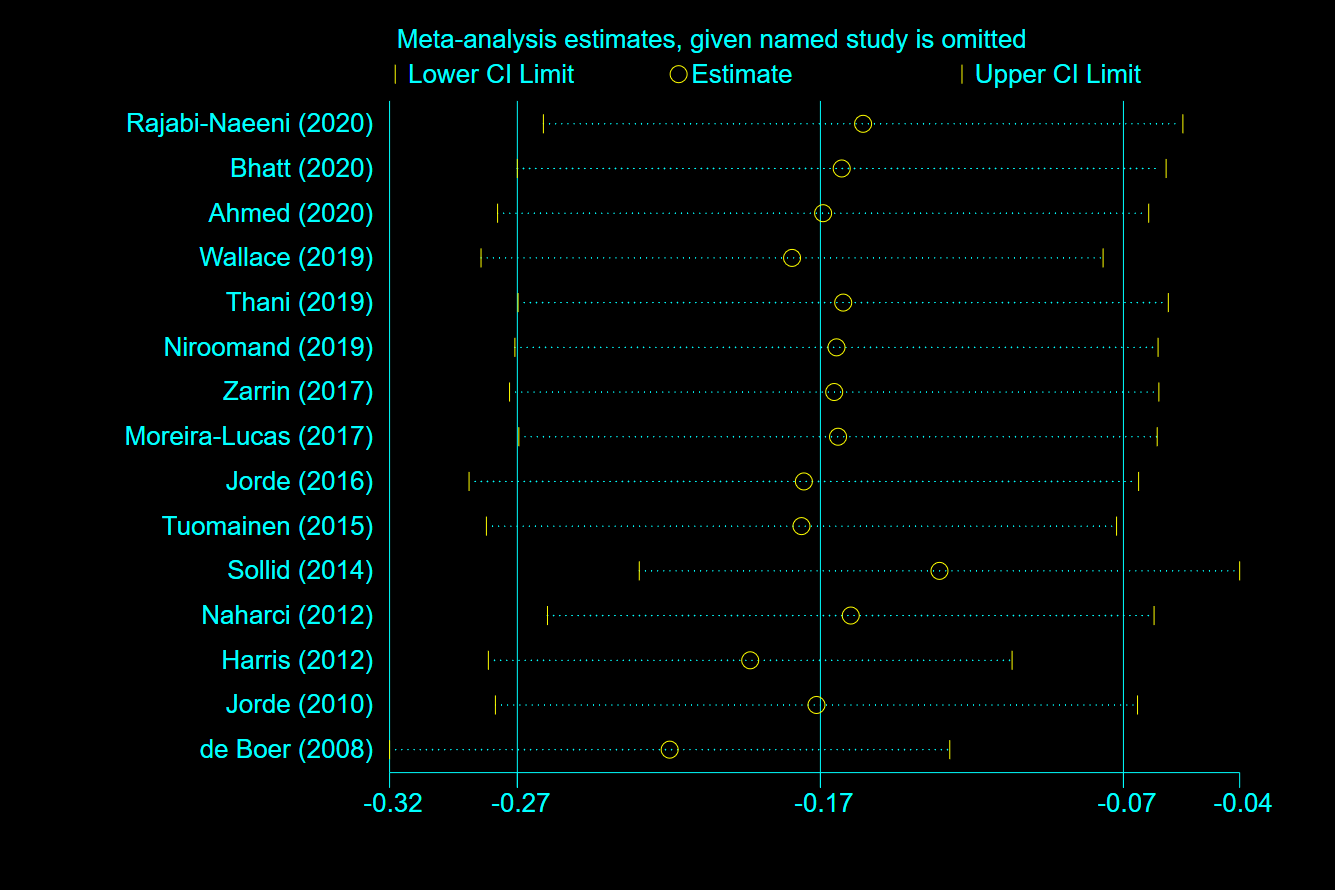


**B**

**
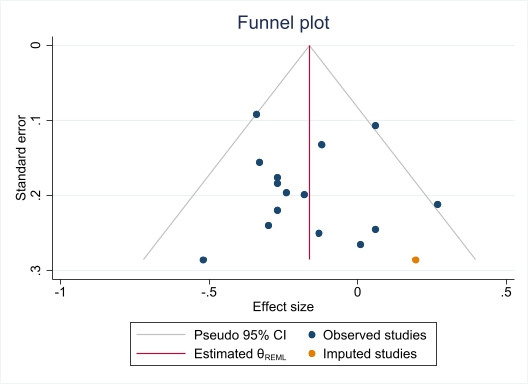
**

**Supplementary Fig. 4**. The results of sensitivity analysis meta-analyses of vitamin D effects on insulin (A) and the trim-and-fill method results (B)

**A**


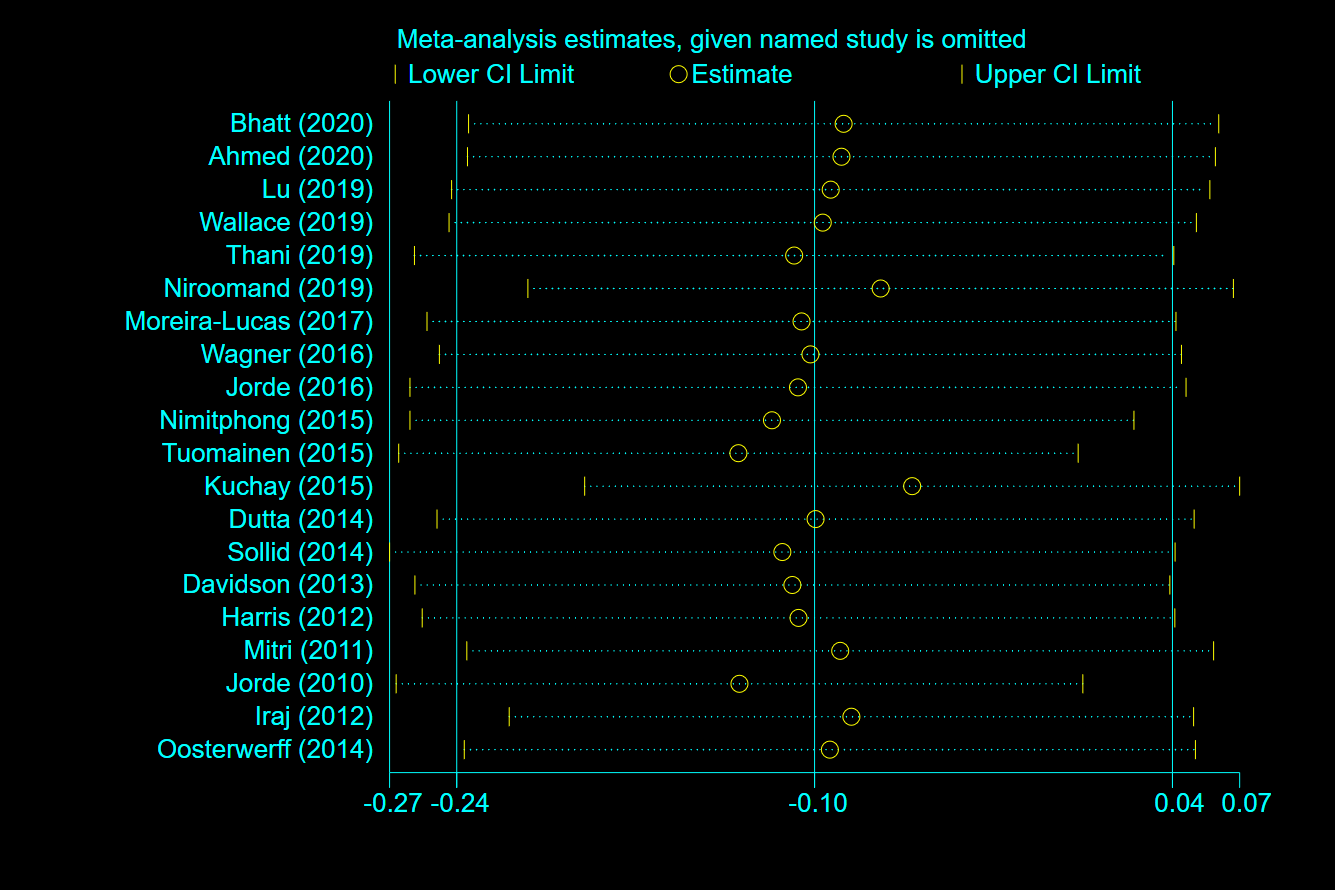


**B**


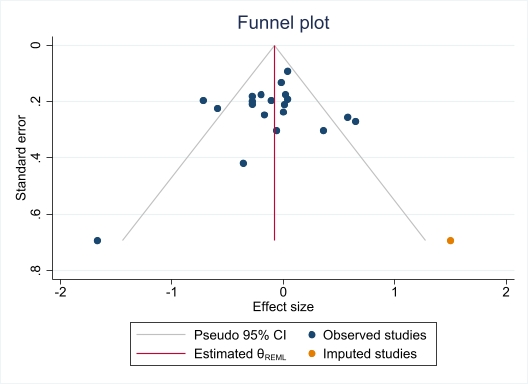


**Supplementary Fig. 5**. The results of sensitivity analysis meta-analyses of vitamin D effects on 2hOGTT (A) and the trim-and-fill method results (B)

**A**


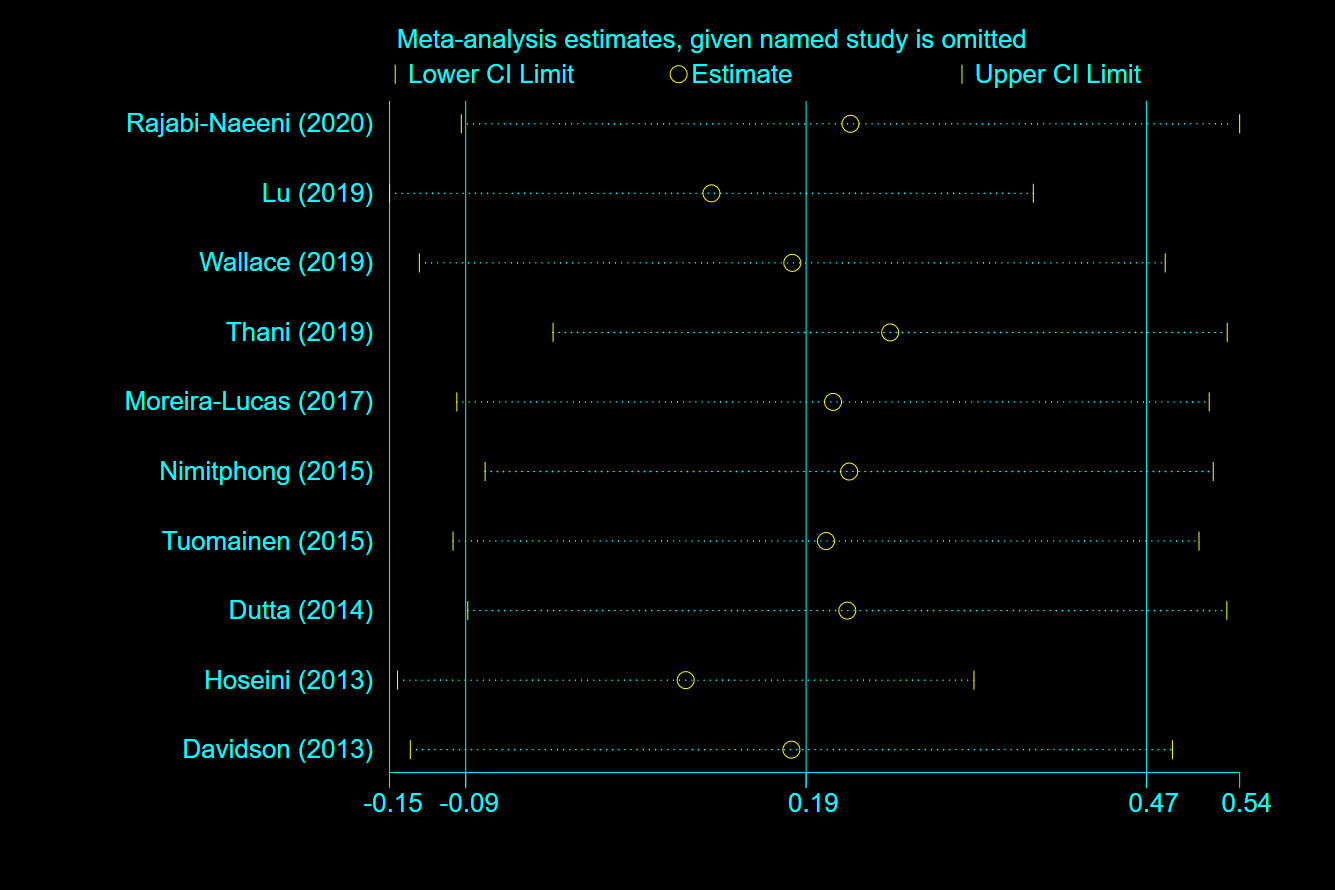


**B**

**
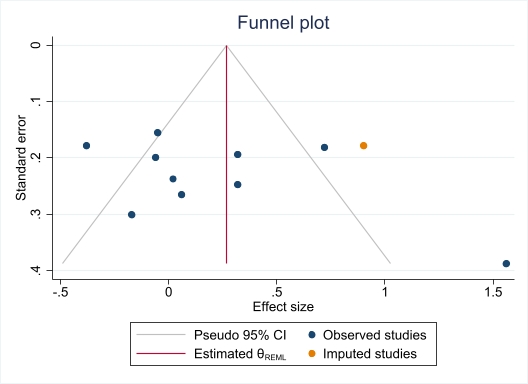
**

**Supplementary Fig. 6**. The results of sensitivity analysis meta-analyses of vitamin D effects on HOMA-B (A) and the trim-and-fill method results (B)

**A**


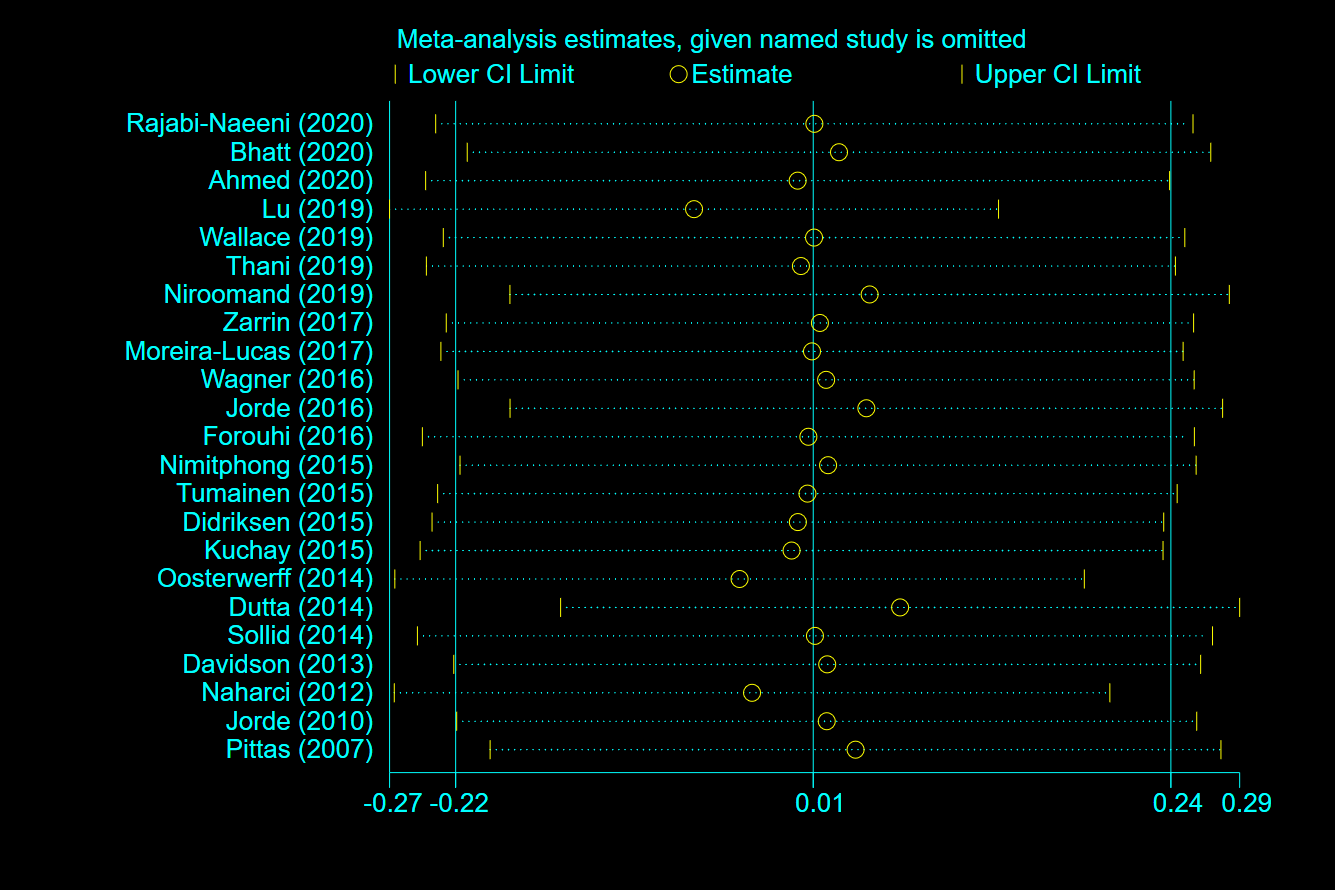


**B**


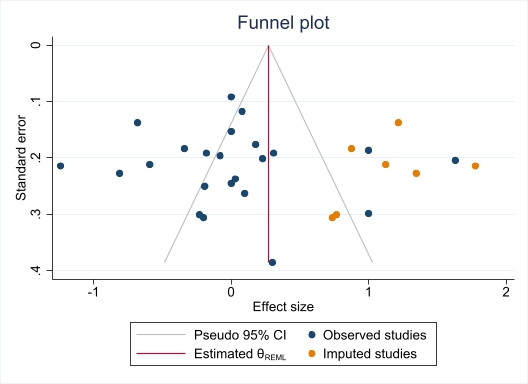
**Supplementary Fig. 7**. The results of sensitivity analysis meta-analyses of vitamin D effects on BMI (A) and the trim-and-fill method results (B)

**A**


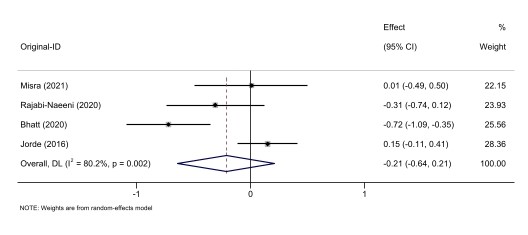


**B**


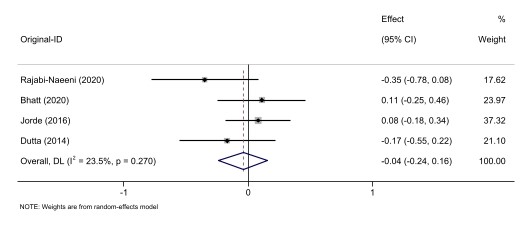


**C**

**
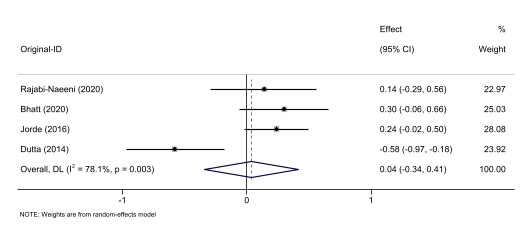
**

**D**


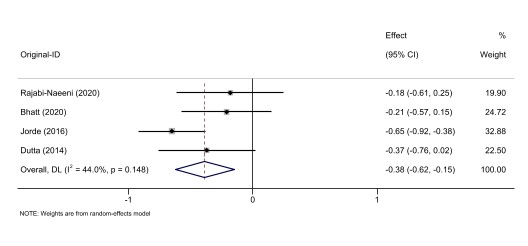


**Supplementary Fig. 8**. Forest plot detailing effect sizes and 95% confidence intervals for the impact of vitamin D supplementation on TC (A), LDL-C (B), HDL-C (C), and TG (D) levels

**A**


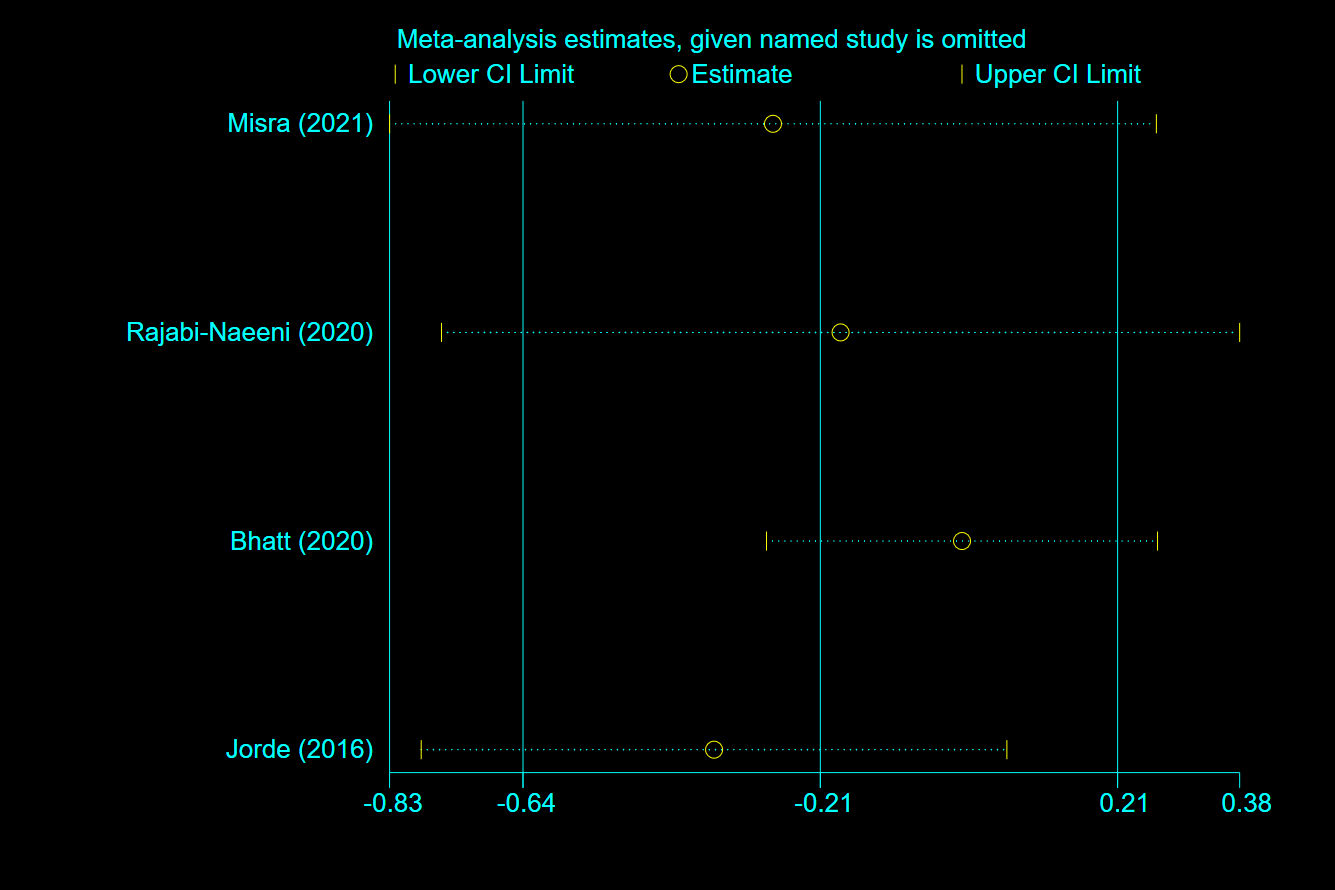


**B**


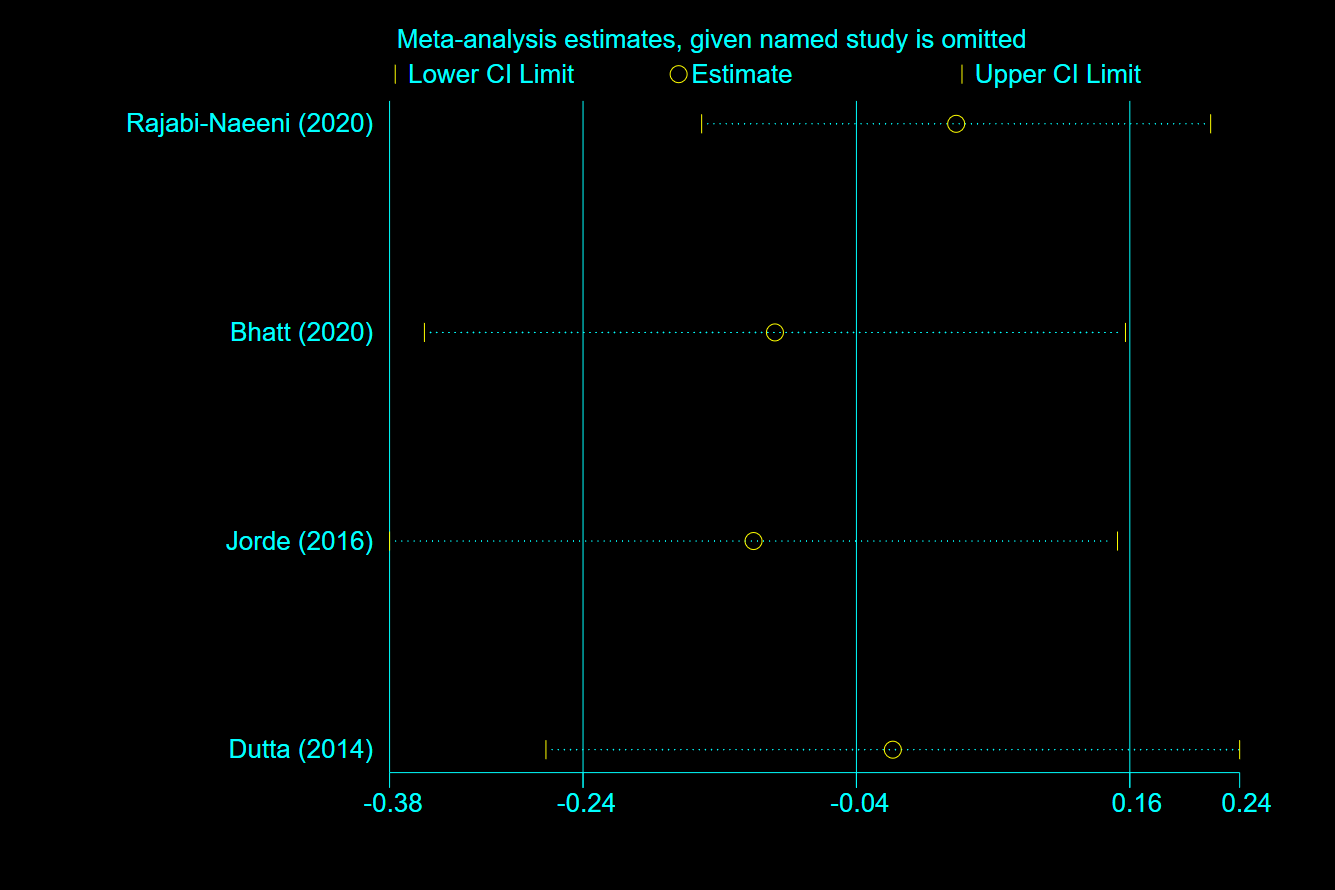


**C**


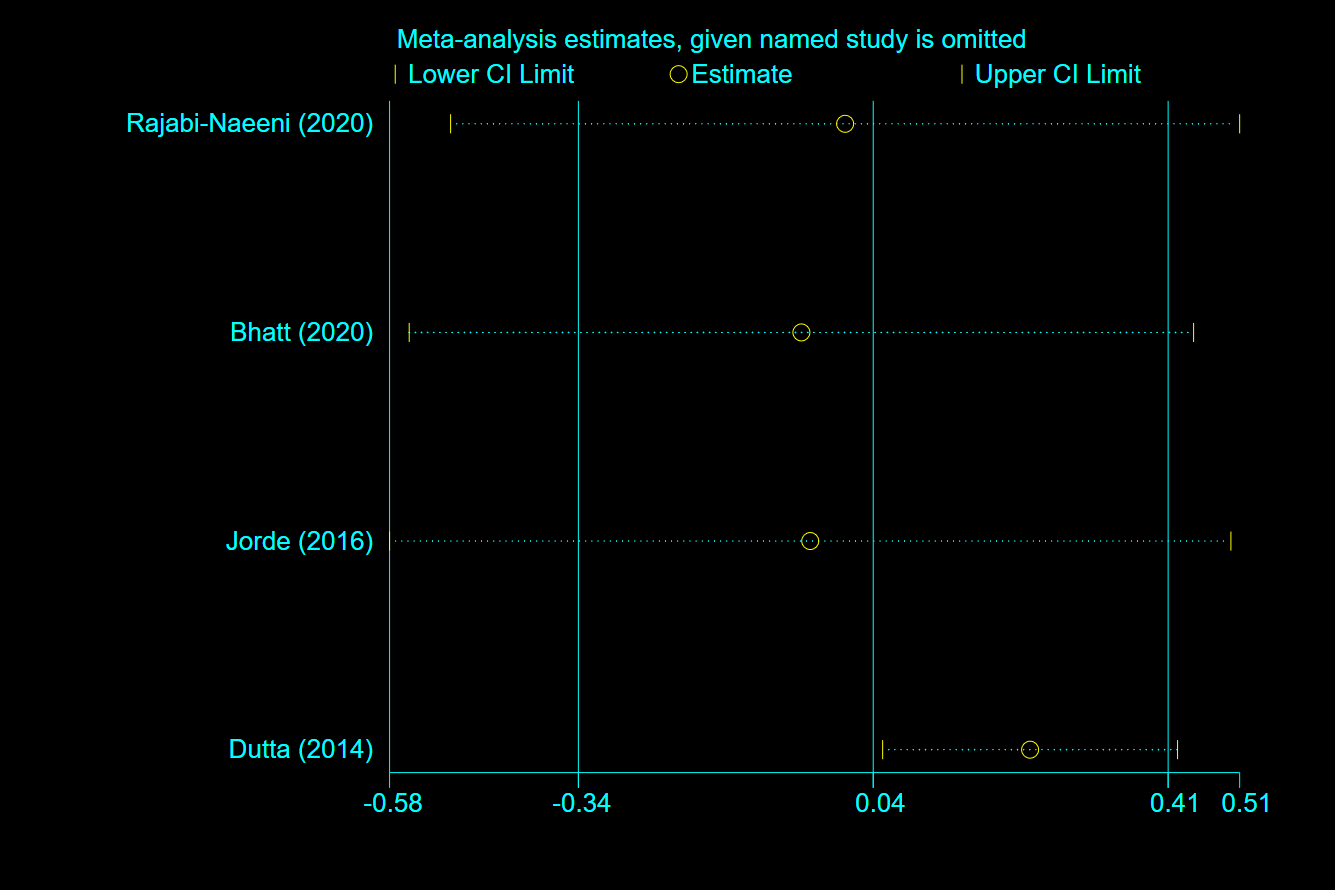


**D**


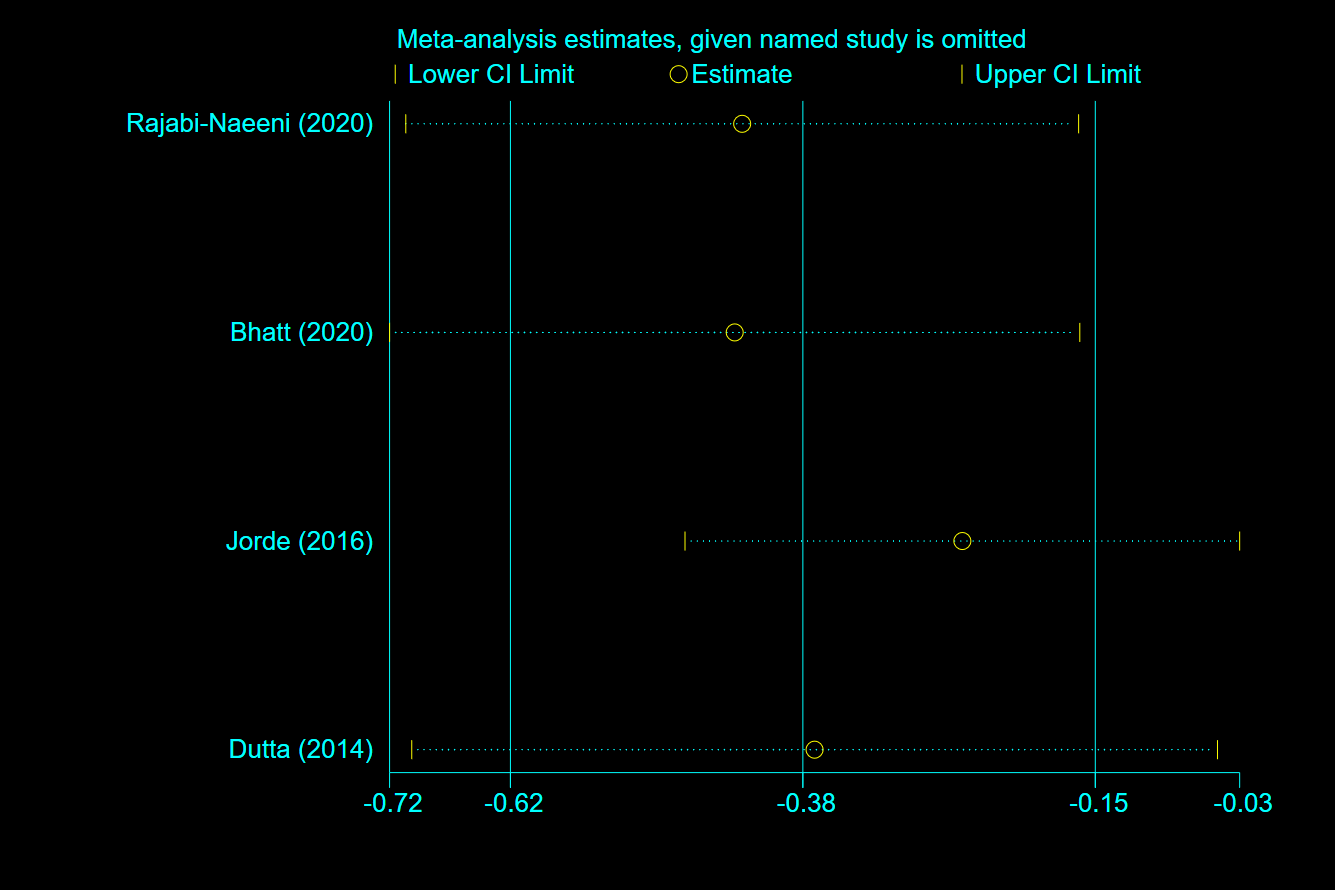


**Supplementary Fig. 9**. The results of sensitivity analysis meta-analyses of vitamin D effects on TC (A), LDL-C (B), HDL-C (C), and TG (D) levels

**A**


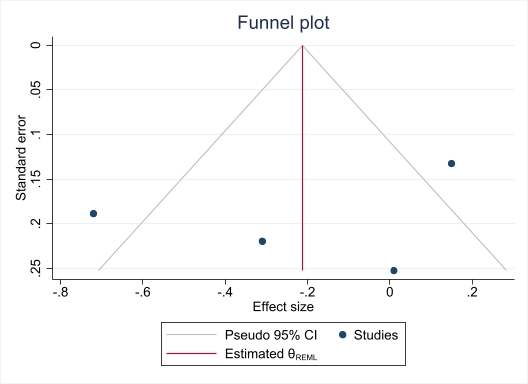


**B**

**
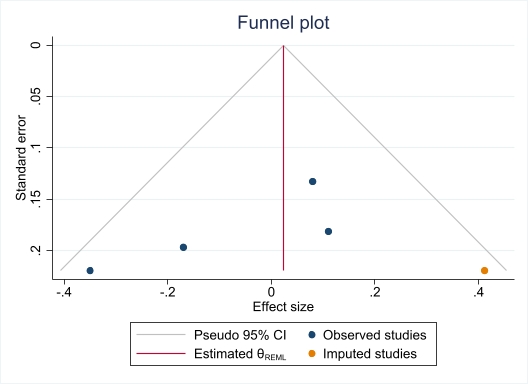
**

**C**

**
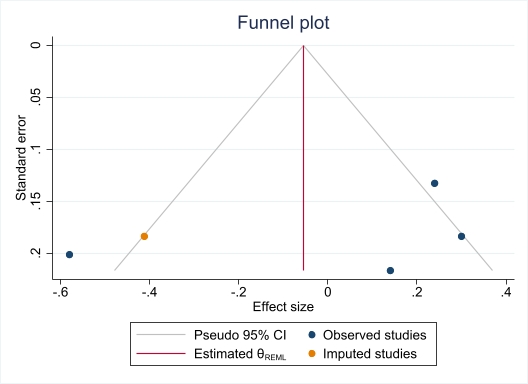
**

**D**


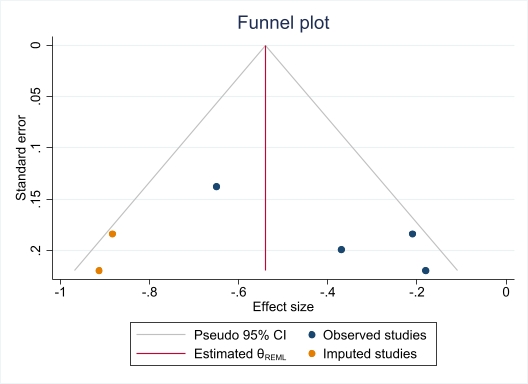


**Supplementary Fig. 10**. The trim-and-fill method results of vitamin D effects on TC (A), LDL-C (B), HDL-C (C), and TG (D) levels

**A**


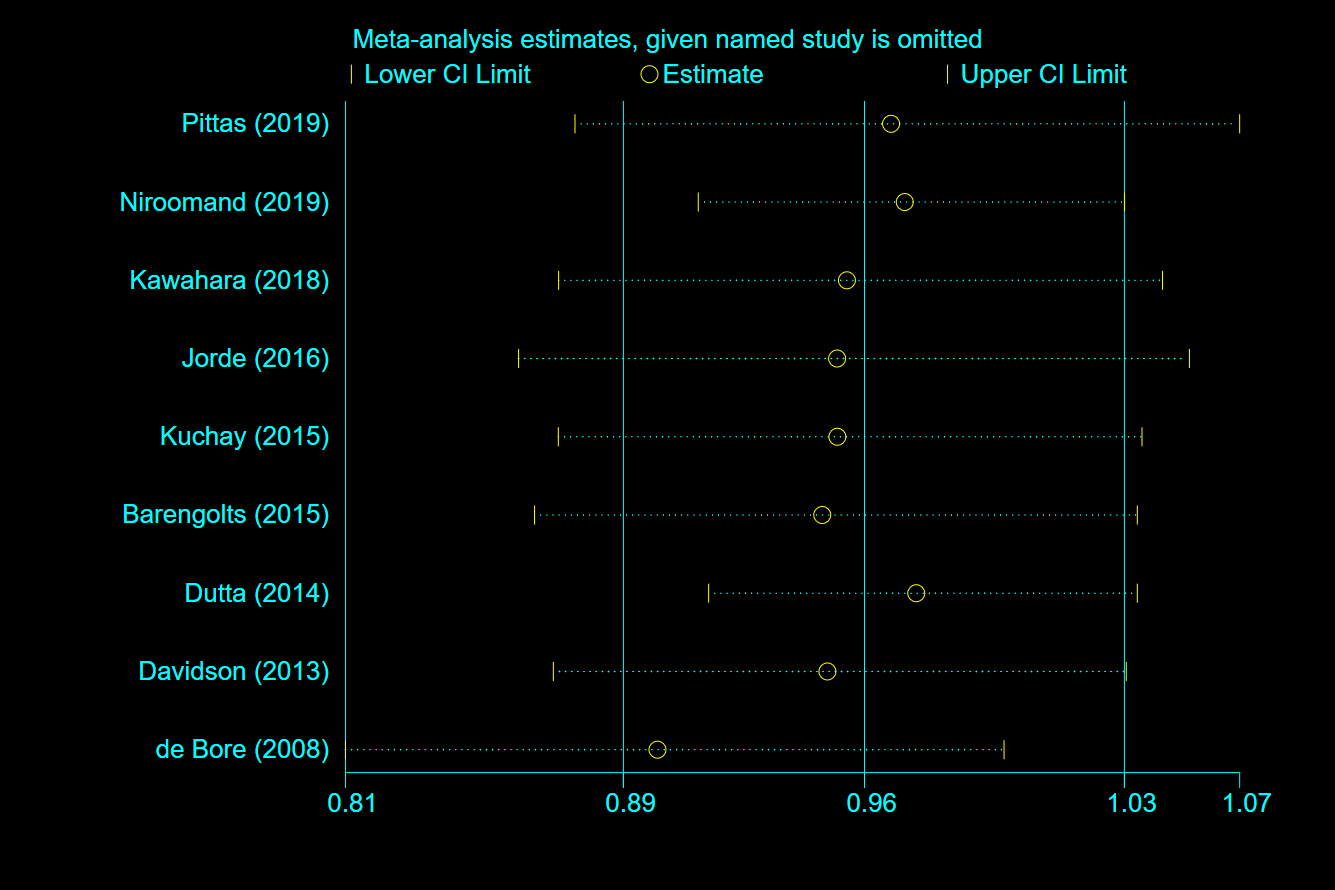


**B**


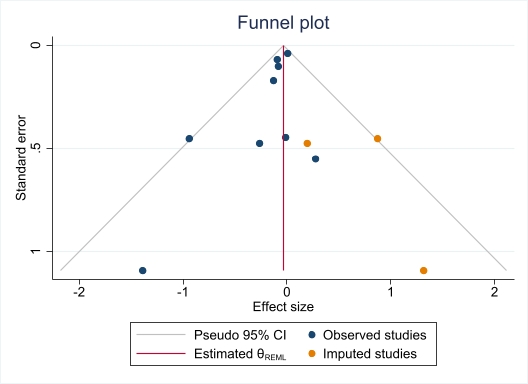


**Supplementary Fig. 11**. The results of sensitivity analysis meta-analyses of vitamin D effects on the risk of diabetes (A) and the trim-and-fill method results (B)
