# Supplementary material for: Effects of vitamin D supplementation on metabolic parameters, anthropometric measures, and diabetes risk in patients with prediabetes: an umbrella review of meta-analyses of randomized controlled trials
Source: Nutr Metab (Lond). 2025 Aug 14;22:99. doi: 10.1186/s12986-025-00994-1 (PMC12351829; doi:10.1186/s12986-025-00994-1)
Supplement: Supplementary file 2 — Supplementary Material 2 [file 12986_2025_994_MOESM2_ESM.docx]

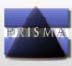


**Supplementary Table 1.** PRISMA 2020 checklist; Effects of vitamin D supplementation on metabolic parameters, anthropometric measures, and diabetes risk in patients with prediabetes: An umbrella review of meta-analyses of randomized controlled trials

| **Section and Topic** | **Item #** | **Checklist item** | **Location where item is reported** |
| --- | --- | --- | --- |
| **TITLE** | | |  |
| Title | 1 | Identify the report as a systematic review. | 1 |
| **ABSTRACT** | | |  |
| Abstract | 2 | See the PRISMA 2020 for Abstracts checklist. | 2, 3 |
| **INTRODUCTION** | | |  |
| Rationale | 3 | Describe the rationale for the review in the context of existing knowledge. | 3 |
| Objectives | 4 | Provide an explicit statement of the objective(s) or question(s) the review addresses. | 4 |
| **METHODS** | | |  |
| Eligibility criteria | 5 | Specify the inclusion and exclusion criteria for the review and how studies were grouped for the syntheses. | 5, 6 |
| Information sources | 6 | Specify all databases, registers, websites, organisations, reference lists and other sources searched or consulted to identify studies. Specify the date when each source was last searched or consulted. | 5 |
| Search strategy | 7 | Present the full search strategies for all databases, registers and websites, including any filters and limits used. | 5, Supplementary Table 2 |
| Selection process | 8 | Specify the methods used to decide whether a study met the inclusion criteria of the review, including how many reviewers screened each record and each report retrieved, whether they worked independently, and if applicable, details of automation tools used in the process. | 5, 6 |
| Data collection process | 9 | Specify the methods used to collect data from reports, including how many reviewers collected data from each report, whether they worked independently, any processes for obtaining or confirming data from study investigators, and if applicable, details of automation tools used in the process. | 5, 6 |
| Data items | 10a | List and define all outcomes for which data were sought. Specify whether all results that were compatible with each outcome domain in each study were sought (e.g. for all measures, time points, analyses), and if not, the methods used to decide which results to collect. | 6 |
|  | 10b | List and define all other variables for which data were sought (e.g. participant and intervention characteristics, funding sources). Describe any assumptions made about any missing or unclear information. | 6 |
| Study risk of bias assessment | 11 | Specify the methods used to assess risk of bias in the included studies, including details of the tool(s) used, how many reviewers assessed each study and whether they worked independently, and if applicable, details of automation tools used in the process. | 6 |
| Effect measures | 12 | Specify for each outcome the effect measure(s) (e.g. risk ratio, mean difference) used in the synthesis or presentation of results. | 7 |
| Synthesis methods | 13a | Describe the processes used to decide which studies were eligible for each synthesis (e.g. tabulating the study intervention characteristics and comparing against the planned groups for each synthesis (item #5)). | 7 |
|  | 13b | Describe any methods required to prepare the data for presentation or synthesis, such as handling of missing summary statistics, or data conversions. | 7 |
|  | 13c | Describe any methods used to tabulate or visually display results of individual studies and syntheses. | 7 |
|  | 13d | Describe any methods used to synthesize results and provide a rationale for the choice(s). If meta-analysis was performed, describe the model(s), method(s) to identify the presence and extent of statistical heterogeneity, and software package(s) used. | 7 |
|  | 13e | Describe any methods used to explore possible causes of heterogeneity among study results (e.g. subgroup analysis, meta-regression). | 7 |
|  | 13f | Describe any sensitivity analyses conducted to assess robustness of the synthesized results. | 7 |
| Reporting bias assessment | 14 | Describe any methods used to assess risk of bias due to missing results in a synthesis (arising from reporting biases). | 7 |
| Certainty assessment | 15 | Describe any methods used to assess certainty (or confidence) in the body of evidence for an outcome. | 6 |
| **RESULTS** | | |  |
| Study selection | 16a | Describe the results of the search and selection process, from the number of records identified in the search to the number of studies included in the review, ideally using a flow diagram. | 7, 8 |
|  | 16b | Cite studies that might appear to meet the inclusion criteria, but which were excluded, and explain why they were excluded. | 7, 8 |
| Study characteristics | 17 | Cite each included study and present its characteristics. | 8 |
| Risk of bias in studies | 18 | Present assessments of risk of bias for each included study. | 8, 9 |
| Results of individual studies | 19 | For all outcomes, present, for each study: (a) summary statistics for each group (where appropriate) and (b) an effect estimate and its precision (e.g. confidence/credible interval), ideally using structured tables or plots. | 9-14 |
| Results of syntheses | 20a | For each synthesis, briefly summarise the characteristics and risk of bias among contributing studies. | 9-14 |
|  | 20b | Present results of all statistical syntheses conducted. If meta-analysis was done, present for each the summary estimate and its precision (e.g. confidence/credible interval) and measures of statistical heterogeneity. If comparing groups, describe the direction of the effect. | 9-14 |
|  | 20c | Present results of all investigations of possible causes of heterogeneity among study results. | 9-14 |
|  | 20d | Present results of all sensitivity analyses conducted to assess the robustness of the synthesized results. | 9-14 |
| Reporting biases | 21 | Present assessments of risk of bias due to missing results (arising from reporting biases) for each synthesis assessed. | 9-14 |
| Certainty of evidence | 22 | Present assessments of certainty (or confidence) in the body of evidence for each outcome assessed. | 9 |
| **DISCUSSION** | | |  |
| Discussion | 23a | Provide a general interpretation of the results in the context of other evidence. | 14, 15 |
|  | 23b | Discuss any limitations of the evidence included in the review. | 16 |
|  | 23c | Discuss any limitations of the review processes used. | 16 |
|  | 23d | Discuss implications of the results for practice, policy, and future research. | 17 |
| **OTHER INFORMATION** | | |  |
| Registration and protocol | 24a | Provide registration information for the review, including register name and registration number, or state that the review was not registered. | 5, 17 |
|  | 24b | Indicate where the review protocol can be accessed, or state that a protocol was not prepared. | 5, 17 |
|  | 24c | Describe and explain any amendments to information provided at registration or in the protocol. | 5 |
| Support | 25 | Describe sources of financial or non-financial support for the review, and the role of the funders or sponsors in the review. | 17 |
| Competing interests | 26 | Declare any competing interests of review authors. | 17 |
| Availability of data, code and other materials | 27 | Report which of the following are publicly available and where they can be found: template data collection forms; data extracted from included studies; data used for all analyses; analytic code; any other materials used in the review. | 17 |

*From:*  Page MJ, McKenzie JE, Bossuyt PM, Boutron I, Hoffmann TC, Mulrow CD, et al. The PRISMA 2020 statement: an updated guideline for reporting systematic reviews. BMJ 2021;372:n71. doi: 10.1136/bmj.n71

For more information, visit: <http://www.prisma-statement.org/>

**Supplementary Table 2.** Effects of vitamin D supplementation on metabolic parameters, anthropometric measures, and diabetes risk in patients with prediabetes: An umbrella review of meta-analyses of randomized controlled trials; Method of the database search strategy using PubMed, Scopus, Cochrane, ScienceDirect, Google Scholar, and Web of Science

| **Database (Search**  **conducted up to**  **July, 2025)** | **Search terms^a^** | **Number of studies searched** |
| --- | --- | --- |
| PubMed | ((((((((((((vitamin D[MeSH Terms]) OR ("vitamin D"[Title/Abstract])) OR (Calciferol[Title/Abstract])) OR ("1,25(OH)2D"[Title/Abstract])) OR ("1,25-dihydroxyvitamin D"[Title/Abstract])) OR (cholecalciferol[Title/Abstract])) OR (ergocalciferol[Title/Abstract])) OR (Hydroxycholecalciferols[Title/Abstract])) OR ("1, 25 dihydroxycholecalciferol"[Title/Abstract])) OR ("1, 25-dihydroxycholecalciferol"[Title/Abstract])) OR ("l,25-(OH)2D3"[Title/Abstract])) OR ("25(OH)D"[Title/Abstract])) AND (((((((((((((prediabetes[MeSH Terms]) OR (prediabetic state[MeSH Terms])) OR (prediabetic states[MeSH Terms])) OR (state, prediabetic[MeSH Terms])) OR (states, prediabetic[MeSH Terms])) OR (prediabetes[Title/Abstract])) OR (prediabetic[Title/Abstract])) OR (pre-diabetes[Title/Abstract])) OR (pre diabetes[Title/Abstract])) OR (pre-diabetic[Title/Abstract])) OR (pre diabetic[Title/Abstract])) OR (pre-diabetics[Title/Abstract])) OR (pre diabetics[Title/Abstract])) | 370 |
| SCOPUS | ( ( TITLE-ABS-KEY ( prediabetes ) OR TITLE-ABS-KEY ( pre-diabetes ) OR TITLE-ABS-KEY ( pre AND diabetes ) OR TITLE-ABS-KEY ( pre AND diabetic ) OR TITLE-ABS-KEY ( pre AND diabetics ) OR TITLE-ABS-KEY ( pre-diabetics ) OR TITLE-ABS-KEY ( pre-diabetic ) OR TITLE-ABS-KEY ( prediabetic ) OR TITLE-ABS-KEY ( prediabetics ) ) ) AND ( ( TITLE-ABS-KEY ( "vitamin D" ) OR TITLE-ABS-KEY ( calciferol ) OR TITLE-ABS-KEY ( "1,25(OH)2D" ) OR TITLE-ABS-KEY ( "25(OH)D" ) OR TITLE-ABS-KEY ( "1,25-dihydroxyvitamin D" ) OR TITLE-ABS-KEY ( cholecalciferol ) OR TITLE-ABS-KEY ( ergocalciferol ) OR TITLE-ABS-KEY ( hydroxycholecalciferols ) OR TITLE-ABS-KEY ( "1, 25 dihydroxycholecalciferol" ) OR TITLE-ABS-KEY ( "1, 25-dihydroxycholecalciferol" ) OR TITLE-ABS-KEY ( "l,25-(OH)2D3" ) ) ) | 1116 |
| Cochrane | #1 (vitamin D):ti,ab,kw OR (Calciferol):ti,ab,kw OR (1 25(OH)2D):ti,ab,kw OR (1 25 dihydroxyvitamin D):ti,ab,kw OR (cholecalciferol):ti,ab,kw (Word variations have been searched) n=18809  #2 (ergocalciferol):ti,ab,kw OR (“1 25 dihydroxycholecalciferol”):ti,ab,kw OR (Hydroxycholecalciferols):ti,ab,kw OR (“1 25 dihydroxycholecalciferol”):ti,ab,kw OR (“l,25(OH)2D3”):ti,ab,kw (Word variations have been searched) n=2071  #3 (prediabetes):ti,ab,kw OR ("pre diabetes"):ti,ab,kw OR ("pre-diabetes"):ti,ab,kw OR ("prediabetic"):ti,ab,kw OR ("pre-diabetic"):ti,ab,kw n=4130  #3 #1 and #3 n=210  #5 #2 and #3 n=23 | 243 |
| Google Scholar | allintitle: “vitamin D” OR Calciferol OR “1,25(OH)2D” OR “1,25-dihydroxyvitamin D” OR cholecalciferol OR ergocalciferol OR Hydroxycholecalciferols OR “1, 25 dihydroxycholecalciferol” OR “1, 25-dihydroxycholecalciferol” OR “l,25-(OH)2D3” OR “25(OH)D” AND prediabetes  allintitle: “vitamin D” OR Calciferol OR “1,25(OH)2D” OR “1,25-dihydroxyvitamin D” OR cholecalciferol OR ergocalciferol OR Hydroxycholecalciferols OR “1, 25 dihydroxycholecalciferol” OR “1, 25-dihydroxycholecalciferol” OR “l,25-(OH)2D3” OR “25(OH)D” AND pre-diabetes  allintitle: “vitamin D” OR Calciferol OR “1,25(OH)2D” OR “1,25-dihydroxyvitamin D” OR cholecalciferol OR ergocalciferol OR Hydroxycholecalciferols OR “1, 25 dihydroxycholecalciferol” OR “1, 25-dihydroxycholecalciferol” OR “l,25-(OH)2D3” OR “25(OH)D” AND prediabetic  allintitle: “vitamin D” OR Calciferol OR “1,25(OH)2D” OR “1,25-dihydroxyvitamin D” OR cholecalciferol OR ergocalciferol OR Hydroxycholecalciferols OR “1, 25 dihydroxycholecalciferol” OR “1, 25-dihydroxycholecalciferol” OR “l,25-(OH)2D3” OR “25(OH)D” AND pre-diabetic  Total | 143  26  11  5  185 |
| ScienceDirect | Title, abstract, keywords: “vitamin D” AND prediabetes  Title, abstract, keywords: "vitamin D" AND pre-diabetes  Title, abstract, keywords: “vitamin D” AND prediabetic  Title, abstract, keywords: "vitamin D" AND pre-diabetic  Title, abstract, keywords: "vitamin D" AND pre-diabetics  Title, abstract, keywords: Calciferol AND prediabetes  Title, abstract, keywords: Calciferol AND prediabetic  Title, abstract, keywords: "1,25(OH)2D" AND prediabetes  Title, abstract, keywords: "1,25(OH)2D" AND pre-diabetes  Title, abstract, keywords: "1,25(OH)2D" AND prediabetic  Title, abstract, keywords: cholecalciferol AND prediabetes  Title, abstract, keywords: cholecalciferol AND pre-diabetes  Title, abstract, keywords: cholecalciferol AND prediabetic  Title, abstract, keywords: “1,25-dihydroxyvitamin D” AND prediabetes  Title, abstract, keywords: “1,25-dihydroxyvitamin D” AND pre-diabetes  Title, abstract, keywords: “1,25-dihydroxyvitamin D” AND prediabetic  Title, abstract, keywords: “1, 25 dihydroxycholecalciferol” AND prediabetes  Title, abstract, keywords: “1, 25 dihydroxycholecalciferol” AND pre-diabetes  Title, abstract, keywords: “1, 25 dihydroxycholecalciferol” AND prediabetic  Title, abstract, keywords: "25(OH)D" AND prediabetes  Title, abstract, keywords: "25(OH)D" AND prediabetic  Total | 63  90  16  23  23  0  0  1  0  0  4  3  1  3  0  2  0  0  0  24  7  259 |
| Web of Sciences | #1 TS=(“vitamin D” OR Calciferol OR “1,25(OH)2D” OR “1,25-dihydroxyvitamin D” OR cholecalciferol OR ergocalciferol OR Hydroxycholecalciferols OR “1, 25 dihydroxycholecalciferol” OR “1, 25-dihydroxycholecalciferol” OR “l,25-(OH)2D3” OR “25(OH)D”)  #2 TS=(prediabetes OR prediabetic OR pre-diabetes OR pre diabetes OR pre-diabetic OR pre-diabetics OR pre diabetic OR pre diabetics)  #1 AND #2 | 133,250  52330  808 |
| Total |  | 2981 |

^a^Searches were limited to original articles, and studies published in the English language using the appropriate filters and/or search terms depending on the database.

**Supplementary Table 3.** The studies excluded via full-text assessment and reasons for exclusions

| **Study** | **Reason of exclusion** |
| --- | --- |
| Pienkowska et al. (1), Lima et al. (2), Pittas et al. (3), Pilz et al. (4) | Being systematic review |
| Ganmaa et al. (5) | Being umbrella review |
| Nimitphong et al. (6) | Studying the effect of vitamin D supplementation on uric acid levels |
| Wimalawansa et al. (7), Barengolts et al. (8) | Narrative review |
| Jayedi et al. (9), Mohammadi et al. (10) | Not relevant outcomes |
| Yutong et al. (11) | Not available full-text |

**Supplementary Table 4**. Summary of the included meta-analyses' findings

| First Author, year of publication/country | No. of included trials | Effect size (Confidence interval) | I^2^ | Certainty of the evidence |
| --- | --- | --- | --- | --- |
| Yang et al. 2023 [40] | 5 | TC: -0.21 (-0.64,0.21) | 81% | NR |
|  |  | TG: -0.42 (-0.59, -0.25) | 45% |  |
|  |  | LDL-C: -0.03 (-0.20, 0.14) | 22% |  |
|  |  | HDL-C: 0.03 (-0.34, 0.41) | 78% |  |
| Zhang et al. 2021 [38] | 29 | BMI: 0.01 (-0.22, 0.24) | 88.7% | NR |
|  |  | 2h-PG: -0.08 (-0.22,0.06) | 56.1% |  |
|  |  | HOMA-IR: 0.15 (0.50, 0.20) | 94.8% |  |
|  |  | FBG: -0.38 (-0.59, -0.16) | 87.6% |  |
|  |  | HbA1c: -0.14 (-0.22, -0.06) | 46.5% |  |
|  |  | HOMA-B: -0.19 (-0.09, 0.47) | 75.9% |  |
|  |  | Insulin: -0.18 (-0.26, -0.09) | 20.7% |  |
| Zou et al. 2021 [39] | 9 | FBS: -2.20 (-3.9, -0.50) | 0.0% | NR |
|  |  | HbA1c: -0.06 (-0.12, 0.01) | 19% |  |
|  |  | 2h-PG: -5.73 (-14.66, 3.20) | 50% |  |
|  |  | Insulin: -13.45 (-25.85, -1.05) | 22% |  |
|  |  | HOMA-IR: 0.38 (0.35, 0.41) | 0.0% |  |
|  |  | BMI: -0.14 (-0.41, -0.13) | 0.0% |  |
|  |  | LDL-C: -0.08 (-0.17, 0.02) | 0.0% |  |
|  |  | HDL-C: 0.07 (0.01, 0.14) | 0.0% |  |
|  |  | TC: -0.05 (-0.78, 0.68) | 86% |  |
|  |  | TG: 0.03 (-0.27, 0.34) | 78% |  |
| Yu et al. 2020 [9] | 8 | FBS: 1.23 (-1.05, 3.51) | 99.2% | NR |
|  |  | HOMA-IR: 0.81 (-2.92, 4.55) | 99.6% |  |
|  |  | HbA1c: 0.01 (-1.08, 1.10) | 98.8% |  |
|  |  | 2h-PG: 1.80 (0.29, 3.31) | 98.6% |  |
| Zhang et al. 2020 [12] | 8 | The risk of T2DM: 0.89 (0.80, 0.90) | 0.0% | NR |
| Barbarawi et al. 2020 [41] | 9 | The risk of T2DM: 0.96 (0.90, 1.03) | 3.0% | NR |
| He et al. 2018 [36] | 28 | FBS: ‑1.76 (‑4.07, 0.55) | <0.1% | NR |
|  |  | HOMA-IR: -0.03 (-0.13, 0.08) | <0.1% |  |
|  |  | The risk of T2DM: 0.86 (0.74, 1.01) | <0.1% |  |
| Mirhosseini et al. 2018 [35] | 28 | FBS: -0.46 (–0.74, -0.19) | 92.4% | NR |
|  |  | HbA1c: -0.48 (-0.79, -0.18) | 92.1% |  |
|  |  | 2h-PG: -0.13 (-0.34, 0.08) | 69.1% |  |
|  |  | HOMA-IR: -0.39 (-0.68, -0.11) | 91.3% |  |
| Poolsup et al. 2016 [26] | 10 | HOMA-IR: -0.06 (-0.36, 0.24) | 1.0% | NR |
|  |  | FBS: -0.10 (-0.18, -0.03) | 18.0% |  |
|  |  | HbA1c: -1 (-2, 0) | 51.0% |  |
|  |  | 2h-PG: -0.23 (0.65, 0.19) | 55.0% |  |
| Seida et al. 2014 [37] | 5 | HbA1c: -0.05 (-0.12, 0.03) | 55.0% | NR |
|  |  | HOMA-IR: -0.04 (-0.30, 0.22) | 45.0% |  |
|  |  | HOMA-β: 1.64 (-25.94, 29.22) | 40.0% |  |
|  |  | The risk of T2DM: 1.02 (0.94, 1.10) | 0.0% |  |
| George et al. 2012 [34] | 14 | FBS: -0.02 (-0.10, 0.06) | 5.0% | NR |
|  |  | HOMA-IR: -0.07 (-0.20, 0.06) | 3.0% |  |
|  |  | HbA1c: 0.03 (-0.18, 0.23) | 0.0% |  |

BMI; Body mass index, FBS; Fasting blood sugar, 2h-PG; 2 h oral glucose tolerance test plasma glucose, HbA1c; Hemoglobin A1c, HOMA-IR; Homeostasis model assessment of insulin resistance, HOMA-β; Homeostasis model assessment of β-cell function, NR; Not reported

**Supplementary Table 5.** Results of the methodological quality assessment of the included meta-analysis

| **Study** | **Q1** | **Q2** | **Q3** | **Q4** | **Q5** | **Q6** | **Q7** | **Q8** | **Q9** | **Q10** | **Q11** | **Q12** | **Q13** | **Q14** | **Q15** | **Q16** | **Quality assessment** |
| --- | --- | --- | --- | --- | --- | --- | --- | --- | --- | --- | --- | --- | --- | --- | --- | --- | --- |
| Yang et al. 2023 | No | No | Yes | Yes | Yes | Yes | Yes | Yes | Yes | Yes | Yes | Yes | Yes | Yes | Yes | Yes | High |
| Pittas et al. 2023 | No | Yes | Yes | Yes | No | No | No | Yes | Yes | Yes | Yes | Yes | Yes | Yes | Yes | Yes | Moderate |
| Zhang et al. 2021 | Yes | No | Yes | Yes | Yes | Yes | Yes | Yes | Yes | No | Yes | Yes | Yes | Yes | Yes | Yes | High |
| Zou et al. 2021 | No | Yes | Yes | Yes | Yes | Yes | Yes | Yes | Yes | Yes | Yes | Yes | Yes | Yes | Yes | Yes | High |
| Yu et al. 2020 | No | No | Yes | Yes | Yes | Yes | Yes | Yes | No | Yes | Yes | Yes | Yes | Yes | Yes | No | Moderate |
| Zhang et al. 2020 | Yes | Yes | Yes | No | Yes | Yes | Yes | Yes | Yes | Yes | Yes | No | Yes | Yes | No | Yes | High |
| Barbarawi et al. 2020 | No | Yes | Yes | Yes | Yes | Yes | Yes | Yes | Yes | Yes | Yes | Yes | Yes | Yes | Yes | Yes | High |
| He et al. 2018 | Yes | No | Yes | No | No | Yes | Yes | Yes | Yes | Yes | Yes | Yes | Yes | No | Yes | Yes | Moderate |
| Mirhosseini et al. 2018 | No | Yes | Yes | No | Yes | Yes | Yes | Yes | Yes | Yes | Yes | Yes | Yes | No | Yes | Yes | High |
| Poolsup et al. 2016 | No | No | Yes | No | Yes | Yes | Yes | Yes | Yes | Yes | Yes | Yes | Yes | No | Yes | Yes | Moderate |
| Seida et al. 2014 | No | No | Yes | Yes | Yes | Yes | Yes | Yes | Yes | Yes | Yes | Yes | Yes | Yes | Yes | Yes | High |
| George et al. 2012 | Partial Yes | No | Yes | No | Yes | Yes | Yes | Yes | Yes | Yes | Yes | Yes | Yes | Yes | No | Yes | Moderate |

* 1. Did the research questions and inclusion criteria for the review include the components of PICO? 2. Did the report of the review contain an explicit statement that the review methods were established prior to the conduct of the review and did the report justify any significant deviations from the protocol? 3. Did the review authors explain their selection of the study designs for inclusion in the review? 4. Did the review authors use a comprehensive literature search strategy? 5. Did the review authors perform study selection in duplicate? 6. Did the review authors perform data extraction in duplicate? 7. Did the review authors provide a list of excluded studies and justify the exclusions? 8. Did the review authors describe the included studies in adequate detail? 9. Did the review authors use a satisfactory technique for assessing the risk of bias (RoB) in individual studies that were included in the review? 10. Did the review authors report on the sources of funding for the studies included in the review? 11. If meta-analysis was performed, did the review authors use appropriate methods for statistical combination of results? 12. If meta-analysis was performed, did the review authors assess the potential impact of RoB in individual studies on the results of the meta-analysis or other evidence synthesis? 13. Did the review authors account for RoB in individual studies when interpreting/ discussing the results of the review? 14. Did the review authors provide a satisfactory explanation for, and discussion of, any heterogeneity observed in the results of the review? 15. If they performed quantitative synthesis, did the review authors carry out an adequate investigation of publication bias (small study bias) and discuss its likely impact on the results of the review? 16. Did the review authors report any potential sources of conflict of interest, including any funding they received for conducting the review?, Each question was answered with “Yes”, “Partial Yes” or “No”. When no meta-analysis was done, question 11, 12 and 15 were answered with “No meta-analysis conducted. Studies with ≥ 13 “yes” answers were categorized as “high”, 9-12 “yes” answers as “moderate”, 5-8 “yes” answers as “low”, and ≤ 4 “yes” answers as “critically low”.

**Supplementary Table 6.** Cochrane Collaboration scale for assessment of quality of the included randomized controlled trials

| **Study ID** | **Selection bias** | | **Performance and detection bias** | **Attrition bias** | **Reporting bias** | **Other bias** | **Overall risk of bias** |
| --- | --- | --- | --- | --- | --- | --- | --- |
|  | **Random sequence generation** | **Allocation concealment** | **Blinding** | **Incomplete outcome data** | **Selective reporting** |  |  |
| Rajabi-Naeeni (2020) | Low | Low | Low | Low | Low | Low | Low |
| Bhatt (2020) | Low | Unclear | Low | Low | Low | Unclear | Low |
| Ansari (2020) | Low | High | Low | Low | Low | Low | Low |
| Ahmed (2020) | Low | Low | Low | Low | Low | Unclear | Low |
| Lu (2019) | Low | Low | Low | Low | Low | Low | Low |
| Wallace (2019) | Unclear | Unclear | Low | Unclear | Low | High | Low |
| Thani (2019) | Unclear | Unclear | Low | Low | Low | Unclear | Low |
| Niroomand (2019) | Low | Unclear | Low | Low | Low | Unclear | Low |
| Zarrin (2017) | Low | Unclear | Low | Low | Low | Low | Low |
| Moreira-Lucas (2017) | Unclear | Low | Low | Low | Low | Low | Low |
| Wagner (2016) | Low | Low | Low | Low | Low | Low | Low |
| Jorde (2016) | Low | Low | Low | Low | Unclear | Low | Low |
| Nimitphong (2015) | Unclear | Low | Low | Low | Low | Low | Low |
| Tuomainen (2015) | Low | Low | Low | Low | Low | Low | Low |
| Kuchay (2015) | Low | High | High | Low | Low | Low | High |
| Dutta (2014) | Low | High | High | Low | Low | Low | High |
| Sollid (2014) | Low | Low | Low | Low | Low | Low | Low |
| Hoseini (2013) | Low | Low | Low | Low | Low | Unclear | Low |
| Davidson (2013) | Low | Low | Low | Low | Low | Low | Low |
| Naharci (2012) | High | Low | Low | Low | Low | Low | Low |
| Harris (2012) | Unclear | Low | Low | Low | Low | Low | Low |
| Mitri (2011) | Low | Low | Low | Low | Low | Low | Low |
| Jorde (2010) | Low | Low | Low | Low | Low | Low | Low |
| de Boer (2008) | Unclear | Low | Low | Low | Low | Low | Low |
| Pittas (2007) | Low | Low | Low | Low | Low | Low | Low |
| Oosterwerff (2014) | Low | Low | Low | Low | Low | Low | Low |
| Iraj (2012) | Low | Low | Low | Low | Low | Unclear | Low |
| Forouhi (2016) | Low | Low | Low | Low | Low | Low | Low |
| Didriksen (2015) | Low | Low | Low | Low | Low | Low | Low |
| Kawahara (2018) | Low | Low | Low | Low | Low | Low | Low |
| Barengolts (2015) | Unclear | Low | Low | Low | Low | Low | Low |

Low, low risk of bias; Unclear, unclear risk of bias; High, High risk of bias

**Supplementary Table 7**. Subgroup analyses for the effects of vitamin D supplementation on prediabetic patients

|  | **Effect size (n)** | **ES (95% CI) ^a^** | **P-within ^b^** | **I^2^ (%) ^c^** | **P-heterogeneity ^d^** |
| --- | --- | --- | --- | --- | --- |
| **Vitamin D on FBS** | | | | | |
| **Publication year** |  |  |  |  |  |
| >2015 | 12 | -0.275 (-0.426, -0.124) | <0.001 | 48.8 | 0.029 |
| ≤2015 | 13 | -0.491 (-0.892, -0.091) | 0.016 | 93.0 | <0.001 |
| **Age (year)** |  |  |  |  |  |
| <50 | 9 | -0.386 (-0.833, 0.060) | 0.090 | 90.7 | <0.001 |
| ≥50 | 12 | -0.419 (-0.735, -0.103) | 0.009 | 89.1 | <0.001 |
| NR | 4 | -0.214 (-0.398, -0.030) | 0.022 | 0.0 | 0.624 |
| **Sample size** |  |  |  |  |  |
| < 100 | 12 | -0.388 (-0.629, -0.147) | 0.107 | 88.7 | <0.001 |
| ≥ 100 | 13 | -0.360 (-0.796, 0.077) | 0.002 | 87.3 | <0.001 |
| **Baseline vitamin D status** |  |  |  |  |  |
| Deficient | 9 | -0.322 (-0.460, -0.183) | <0.001 | 21.9 | 21.9 |
| Sufficient and insufficient | 7 | -0.485 (-1.142, 0.173) | 0.149 | <0.001 | 93.9 |
| Insufficient and deficient | 5 | -0.016 (-0.227, 0.195) | 0.882 | 0.838 | 0.0 |
| Sufficient, insufficient, and deficient | 2 | -1.210 (-3.268, 0.848) | 0.249 | <0.001 |  |
| NR | 2 | -0.323 (-0.929, 0.284) | 0.297 | 0.003 |  |
| **Dosage (IU/week)** |  |  |  |  |  |
| < 30000 | 15 | -0.406 (-0.665, -0.147) | 0.002 | 87.1 | <0.001 |
| ≥ 30000 | 10 | -0.326 (-0.721, 0.068) | 0.105 | 83.57 | <0.001 |
| **Intervention duration (weeks)** |  |  |  |  |  |
| ≤ 24 | 14 | -0.189 (-0.356, -0.021) | 0.028 | 52.2 | <0.001 |
| > 24 | 11 | -0.646 (-1.044, -0.248) | 0.001 | 93.8 | <0.001 |
| **Vitamin D on HbA1C** | | | | | |
| **Publication year** |  |  |  |  |  |
| >2015 | 13 | -0.500 (-0.923, -0.076) | 0.021 | 94.7 | <0.001 |
| ≤2015 | 15 | -0.454 (-0.749, -0.160) | 0.002 | 92.7 | <0.001 |
| **Age (year)** |  |  |  |  |  |
| <50 | 10 | -0.406 (-0.812, 0.000) | 0.050 | 89.9 | <0.001 |
| ≥50 | 14 | -0.608 (-0.986, -0.230) | 0.002 | 96.3 | <0.001 |
| NR | 4 | -0.214 (-0.398, -0.030) | 0.022 | 0.0 | 0.624 |
| **Sample size** |  |  |  |  |  |
| < 100 | 13 | -0.377 (-0.773, 0.019) | 0.001 | 87.9 | <0.001 |
| ≥ 100 | 15 | -0.560 (-0.877, -0.242) | 0.062 | 96.1 | <0.001 |
| **Baseline vitamin D status** |  |  |  |  |  |
| Deficient | 10 | -0.274 (-0.453, -0.094) | 0.003 | 68.8 | 0.001 |
| Sufficient and insufficient | 8 | -0.444 (-1.030, 0.141) | 0.137 | 92.9 | <0.001 |
| Insufficient and deficient | 6 | -0.118 (-0.255, 0.018) | 0.089 | 0.0 | 0.617 |
| Sufficient, insufficient, and deficient | 2 | -2.798 (-3.817, -1.779) | <0.001 | 90.5 | 0.001 |
| NR | 2 | -0.323 (-0.929, 0.284) | 0.297 | 88.5 | 0.003 |
| **Dosage (IU/week)** |  |  |  |  |  |
| < 30000 | 15 | -0.373 (-0.614, -0.131) | 0.003 | 96.6 | <0.001 |
| ≥ 30000 | 13 | -0.595 (-1.077, -0.113) | 0.016 | 86.3 | <0.001 |
| **Intervention duration (weeks)** |  |  |  |  |  |
| ≤ 24 | 16 | -0.395 (-0.816, 0.025) | 0.065 | 93.8 | <0.001 |
| > 24 | 12 | -0.569 (-0.872, -0.265) | <0.001 | 94.3 | <0.001 |
| NR |  |  |  |  |  |
| **Vitamin D on HOMA-IR** | | | | | |
| **Publication year** |  |  |  |  |  |
| >2015 | 9 | 0.140 (-0.542, 0.823) | 0.687 | 96.6 | <0.001 |
| ≤2015 | 13 | -0.341 (-0.727, 0.045) | 0.083 | 91.8 | <0.001 |
| **Age (year)** |  |  |  |  |  |
| <50 | 8 | -0.196 (-0.351, -0.042) | 0.013 | 9.4 | 0.358 |
| ≥50 | 10 | -0.604 (-1.074, -0.134) | 0.012 | 94.5 | <0.001 |
| NR | 4 | 1.048 (-0.563, 2.659) | 0.202 | 98.1 | <0.001 |
| **Sample size** |  |  |  |  |  |
| < 100 | 10 | -0.390 (-0.984, 0.203) | 0.197 | 96.1 | <0.001 |
| ≥ 100 | 12 | 0.034 (-0.424, 0.492) | 0.883 | 91.2 | <0.001 |
| **Baseline vitamin D status** |  |  |  |  |  |
| Deficient | 10 | 0.293 (-0.332, 0.918) | 0.358 | 95.2 | <0.001 |
| Sufficient and insufficient | 8 | -0.739 (-1.344, -0.135) | 0.017 | 94.4 | <0.001 |
| Insufficient | 3 | \| -0.076 (-0.279, 0.128) | 0.466 | 0.0 | 0.444 |
| NR | 1 | 0.050 (-0.165, 0.2650) | 0.649 | - | - |
| **Dosage (IU/week)** |  |  |  |  |  |
| < 30000 | 13 | -0.478 (-0.858, -0.098) | 0.014 | 93.2 | <0.001 |
| ≥ 30000 | 9 | 0.384 (-0.329, 1.096) | 0.291 | 95.7 | <0.001 |
| **Intervention duration (weeks)** |  |  |  |  |  |
| ≤ 24 | 11 | -0.181 (-0.340, -0.023) | 0.025 | 23.4 | 0.221 |
| > 24 | 11 | -0.143 (-0.755, 0.469) | 0.647 | 97.3 | <0.001 |
| **Vitamin D on insulin** | | | | | |
| **Publication year** |  |  |  |  |  |
| >2015 | 9 | -0.217 (-0.337, -0.097) | <0.001 | 0.0 | 0.950 |
| ≤2015 | 6 | -0.102 (-0.337, 0.132) | 0.393 | 64.5 | 0.015 |
| **Age (year)** |  |  |  |  |  |
| <50 | 7 | -0.270 (-0.411, -0.128) | <0.001 | 0.0 | 0.999 |
| ≥50 | 7 | -0.088 (-0.279, 0.104) | 0.370 | 59.1 | 0.023 |
| NR | 1 | -0.130 (-0.620, 0.360) | 0.603 | - | - |
| **Sample size** |  |  |  |  |  |
| < 100 | 7 | -0.105 (-0.301, 0.091) | 0.293 | 15.8 | 0.310 |
| ≥ 100 | 8 | -0.200 (-0.317, -0.082) | 0.001 | 27.3 | 0.211 |
| **Baseline vitamin D status** |  |  |  |  |  |
| Deficient | 5 | -0.266 (-0.430, -0.102) | 0.001 | 0.0 | 0.985 |
| Sufficient and insufficient | 4 | -0.277 (-0.461, -0.094) | 0.003 | 11.7 | 0.335 |
| Insufficient and deficient | 4 | -0.081 (-0.354, 0.192) | 0.560 | 38.1 | 0.183 |
| NR | 2 | -0.013 (-0.186, 0.160) | 0.883 | 10.3 | 0.291 |
| **Dosage (IU/week)** |  |  |  |  |  |
| < 30000 | 10 | -0.124 (-0.269, 0.021) | 0.094 | 41.8 | 0.079 |
| ≥ 30000 | 5 | -0.271 (-0.432, -0111) | 0.001 | 0.0 | 0.986 |
| **Intervention duration (weeks)** |  |  |  |  |  |
| < 24 | 5 | -0.125 (-0.337, 0.087) | 0.249 | 31.7 | 0.210 |
| ≥ 24 | 10 | -0.189 (-0.307, -0.072) | 0.002 | 22.2 | 0.239 |
| **Vitamin D on 2h-PG** | | | | | |
| **Publication year** |  |  |  |  |  |
| >2015 | 9 | -0.156 (-0.282, -0.030) | 0.015 | 0.0 | 0.511 |
| ≤2015 | 11 | -0.034 (-0.279, 0.210) | 0.784 | 71.1 | <0.001 |
| **Age (year)** |  |  |  |  |  |
| <50 | 8 | -0.306 (-0.574, -0.038) | 0.025 | 61.2 | 0.012 |
| ≥50 | 9 | -0.000 (-0.145, 0.145) | 1.000 | 27.2 | 0.202 |
| NR | 3 | 0.034 (-0.432, 0.500) | 0.886 | 74.3 | 0.020 |
| **Sample size** |  |  |  |  |  |
| < 100 | 10 | -0.020 (-0.306, 0.267) | 0.892 | 67.0 | 0.001 |
| ≥ 100 | 10 | -0.146 (-0.291, -0.002) | 0.047 | 44.3 | 0.064 |
| **Baseline vitamin D status** |  |  |  |  |  |
| Deficient | 7 | -0.305 (-0.519, -0.090) | 0.005 | 31.4 | 0.188 |
| Sufficient and insufficient | 4 | 0.086 (-0.180, 0.353) | 0.525 | 42.5 | 0.156 |
| Insufficient and deficient | 5 | 0.132 (-0.121, 0.386) | 0.306 | 37.4 | 0.172 |
| Sufficient, insufficient, deficient | 3 | -0.236 (-0.683, 0.212) | 0.302 | 79.6 | 0.007 |
| **Dosage (IU/week)** |  |  |  |  |  |
| < 30000 | 11 | 0.035 (-0.120, 0.190) | 0.658 | 40.0 | 0.082 |
| ≥ 30000 | 9 | -0.280 (-0.495, -0.064) | 0.011 | 55.1 | 0.023 |
| **Intervention duration (weeks)** |  |  |  |  |  |
| < 24 | 8 | -0.066 (-0.372, 0.240) | 0.673 | 59.2 | 0.016 |
| ≥ 24 | 12 | -0.117 (-0.277, 0.042) | 0.150 | 59.3 | 0.005 |
| **Vitamin D on HOMA-B** | | | | | |
| **Publication year** |  |  |  |  |  |
| >2015 | 5 | 0.121 (-0.269, 0.511) | 0.543 | 80.7 | <0.001 |
| ≤2015 | 5 | 0.281 (-0.170, 0.732) | 0.222 | 75.3 | 0.003 |
| **Age (year)** |  |  |  |  |  |
| <50 | 3 | 0.529 (-0.196, 1.254) | 0.153 | 85.6 | 0.001 |
| ≥50 | 6 | 0.103 (-0.268, 0.474) | 0.586 | 75.8 | 0.001 |
| NR | 1 | -0.050 (-0.355, 0.255) | 0.748 | - | - |
| **Sample size** |  |  |  |  |  |
| < 100 | 5 | 0.306 (-0.168, 0.780) | 0.206 | 73.1 | 0.005 |
| ≥ 100 | 5 | 0.108 (-0.262, 0.478) | 0.567 | 81.7 | <0.001 |
| **Baseline vitamin D status** |  |  |  |  |  |
| Deficient | 4 | 0.029 (-0.168, 0.227) | 0.770 | 4.7 | 0.369 |
| Sufficient and insufficient | 2 | 0.904 (-0.309, 2.117) | 0.144 | 86.2 | 0.007 |
| Insufficient and deficient | 3 |  | 0.329 | 27.9 | 0.250 |
| NR | 1 | 0.720 (0.365, 1.075) | <0.001 | - | - |
| **Dosage (IU/week)** |  |  |  |  |  |
| < 30000 | 5 | 0.227 (-0.109, 0.563) | 0.185 | 60.1 | 0.040 |
| ≥ 30000 | 5 | 0.183 (-0.251, 0.617) | 0.409 | 83.0 | <0.001 |
| **Intervention duration (weeks)** |  |  |  |  |  |
| < 24 | 4 | 0.156 (-0.181, 0.492) | 0.346 | 81.2 | 0.001 |
| ≥ 24 | 6 | 0.286 (-0.308, 0.879) | 0.365 | 76.6 | 0.001 |
| **Vitamin D on BMI** | | | | | |
| **Publication year** |  |  |  |  |  |
| >2015 | 12 | 0.004 (-0.323, 0.331) | 0.982 | 89.8 | <0.001 |
| ≤2015 | 11 | 0.016 (-0.343, 0.375) | 0.932 | 88.5 | <0.001 |
| **Age (year)** |  |  |  |  |  |
| <50 | 9 | 0.083 (-0.214, 0.381) | 0.583 | 82.1 | <0.001 |
| ≥50 | 11 | 0.118 (-0.253, 0.489) | 0.534 | 90.8 | <0.001 |
| NR | 2 | -0.723 (-1.752, 0.306) | 0.168 | 90.2 | 0.001 |
| **Sample size** |  |  |  |  |  |
| < 100 | 10 | -0.086 (-0.391, 0.219) | 0.582 | 70.9 | <0.001 |
| ≥ 100 | 13 | 0.067 (-0.252, 0.387) | 0.679 | 92.5 | <0.001 |
| **Baseline vitamin D status** |  |  |  |  |  |
| Deficient | 7 | -0.020 (-0.413, 0.374) | 0.921 | 87.3 | <0.001 |
| Sufficient and insufficient | 5 | 0.171 (-0.176, 0.517) | 0.335 | 66.1 | 0.019 |
| Insufficient and deficient | 5 | -0.234 (-0.795, 0.327) | 0.414 | 86.8 | <0.001 |
| Sufficient, insufficient, and deficient | 3 | -0.152 (-0.739, 0.435) | 0.611 | 80.1 | 0.007 |
| NR | 3 | 0.333 (-0.779, 1.444) | 0.557 | 97.7 | <0.001 |
| **Dosage (IU/week)** |  |  |  |  |  |
| < 30000 | 13 | 0.170 (-0.197, 0.538) | 0.197 | 91.2 | <0.001 |
| ≥ 30000 | 10 | -0.183 (-0.461, 0.095) | 0.364 | 82.9 | <0.001 |
| **Intervention duration (weeks)** |  |  |  |  |  |
| < 24 | 6 | 0.003 (-0.169, 0.175) | 0.974 | 0.0 | 0.758 |
| ≥ 24 | 17 | 0.021 (-0.281, 0.323) | 0.890 | 91.7 | <0.001 |
| **Vitamin D on diabetes risk** | | | | | |
| **Publication year** |  |  |  |  |  |
| >2015 | 4 | 0.907 (0.815, 1.010) | 0.074 | 0.0 | 0.697 |
| ≤2015 | 5 | 0.927 (0.705, 1.218) | 0.584 | 18.5 | 0.297 |
| **Age (year)** |  |  |  |  |  |
| <50 | 3 | 0.849 (0.434, 1.661) | 0.632 | 0.0 | 0.381 |
| ≥50 | 4 | 0.978 (0.917, 1.043) | 0.490 | 0.0 | 0.560 |
| NR | 2 | 0.653 (0.303, 1.408) | 0.277 | 64.3 | 0.094 |
| **Sample size** |  |  |  |  |  |
| < 150 | 4 | 0.641 (0.345, 1.193) | 0.160 | 21.0 | 0.284 |
| ≥ 150 | 4 | 0.978 (0.917, 1.043) | 0.490 | 0.0 | 0.560 |
| NR | 1 | 0.880 (0.627, 1.235) | 0.459 | - | - |
| **Baseline vitamin D status** |  |  |  |  |  |
| Deficient | 3 | 0.968 (0.506, 1.853) | 0.922 | 0.0 | 0.397 |
| Sufficient and insufficient | 3 | 0.749 (0.466, 1.204) | 0.232 | 42.4 | 0.176 |
| NR | 3 | 0.978 (0.912, 1.049) | 0.539 | 3.4 | 0.355 |
| **Dosage (IU/week)** |  |  |  |  |  |
| < 30000 | 3 | 0.976 (0.913, 1.043) | 0.480 | 2.8 | 0.358 |
| ≥ 30000 | 5 | 0.720 (0.441, 1.175) | 0.189 | 10.8 | 0.344 |
| NR | 1 | 0.880 (0.627, 1.235) | 0.459 | - | - |
| **Intervention duration (weeks)** |  |  |  |  |  |
| ≤ 12 | 4 | 0.898 (0.527, 1.531) | 0.693 | 0.0 | 0.572 |
| > 12 | 4 | 0.941 (0.834, 1.062) | 0.324 | 51.0 | 0.106 |
| NR | 1 | 0.880 (0.627, 1.235) | 0.459 | - | - |

ES, Effect size; CI, confidence interval.

^a^Obtained from the Random-effects model.

^b^Refers to the mean (95% CI).

^c^Inconsistency, percentage of variation across studies due to heterogeneity.

^d^Obtained from the Q-test.

**References:**

1. Pieńkowska A, Janicka J, Duda M, Dzwonnik K, Lip K, Mędza A, et al. Controversial Impact of Vitamin D Supplementation on Reducing Insulin Resistance and Prevention of Type 2 Diabetes in Patients with Prediabetes: A Systematic Review. Nutrients. 2023;15(4).

2. Lima CHR, Layanne L, Nogueira NDN, Rodrigues GP, Frota KMG, Teixeira N, et al. Effects of vitamin D supplementation on the glycemic control of pre-diabetic individuals: a systematic review. Nutricion hospitalaria. 2021;38(1):186-93.

3. Pittas AG, Jorde R, Kawahara T, Dawson-Hughes B. Vitamin D Supplementation for Prevention of Type 2 Diabetes Mellitus: To D or Not to D? The Journal of clinical endocrinology and metabolism. 2020;105(12):3721-33.

4. Pilz S, Kienreich K, Rutters F, de Jongh R, van Ballegooijen AJ, Grübler M, et al. Role of vitamin D in the development of insulin resistance and type 2 diabetes. Current diabetes reports. 2013;13(2):261-70.

5. Ganmaa D, Enkhmaa D, Nasantogtokh E, Sukhbaatar S, Tumur-Ochir KE, Manson JE. Vitamin D, respiratory infections, and chronic disease: Review of meta-analyses and randomized clinical trials. Journal of internal medicine. 2022;291(2):141-64.

6. Nimitphong H, Saetung S, Chailurkit LJJoC, Endocrinology T. or, Chanprasertyothin, S., & Ongphiphadhanakul, B.(2021). Vitamin D supplementation is associated with serum uric acid concentration in patients with prediabetes and hyperuricemia.24:100255.

7. Wimalawansa SJ. Associations of vitamin D with insulin resistance, obesity, type 2 diabetes, and metabolic syndrome. The Journal of steroid biochemistry and molecular biology. 2018;175:177-89.

8. Barengolts E. Vitamin D and prebiotics may benefit the intestinal microbacteria and improve glucose homeostasis in prediabetes and type 2 diabetes. Endocrine practice : official journal of the American College of Endocrinology and the American Association of Clinical Endocrinologists. 2013;19(3):497-510.

9. Jayedi A, Daneshvar M, Jibril AT, Sluyter JD, Waterhouse M, Romero BD, et al. Serum 25(OH)D Concentration, Vitamin D Supplementation, and Risk of Cardiovascular Disease and Mortality in Patients with Type 2 Diabetes or Prediabetes: a Systematic Review and Dose-Response Meta-Analysis. The American journal of clinical nutrition. 2023;118(3):697-707.

10. Mohammadi S, Hajhashemy Z, Saneei P. Serum vitamin D levels in relation to type-2 diabetes and prediabetes in adults: a systematic review and dose-response meta-analysis of epidemiologic studies. Critical reviews in food science and nutrition. 2022;62(29):8178-98.

11. Yutong Z, Bo G, Songlin Y, Danchen W, Ling Q. Effect of vitamin d supplementation on glycose homeostasis, islet function and common metabolic indexes in diabetes and prediabetes: A systematic review and meta-analysis. Clinica Chimica Acta. 2022;530:S372-S3.
